# Suppression of Plant Immune Responses by the *Pseudomonas savastanoi* pv. *savastanoi* NCPPB 3335 Type III Effector Tyrosine Phosphatases HopAO1 and HopAO2

**DOI:** 10.3389/fpls.2017.00680

**Published:** 2017-05-05

**Authors:** María Pilar Castañeda-Ojeda, Alba Moreno-Pérez, Cayo Ramos, Emilia López-Solanilla

**Affiliations:** ^1^Área de Genética, Facultad de Ciencias, Instituto de Hortofruticultura Subtropical y Mediterránea “La Mayora”, Universidad de Málaga – Consejo Superior de Investigaciones CientíficasMálaga, Spain; ^2^Centro de Biotecnología y Genómica de Plantas, Universidad Politécnica de Madrid – Instituto Nacional de Investigación y Tecnología Agraria y Alimentaria, Parque Científico y Tecnológico de la UPMMadrid, Spain; ^3^Departamento de Biotecnología y Biología Vegetal, Escuela Técnica Superior de Ingeniería Agronómica, Alimentaria y de Biosistemas, Universidad Politécnica de MadridMadrid, Spain

**Keywords:** HopAO, T3E, T3SS, *Pseudomonas savastanoi*, *Pseudomonas syringae*, olive plant, plant immunity

## Abstract

The effector repertoire of the olive pathogen *P. savastanoi* pv. *savastanoi* NCPPB 3335 includes two members of the HopAO effector family, one of the most diverse T3E families of the *P. syringae* complex. The study described here explores the phylogeny of these dissimilar members, HopAO1 and HopAO2, among the complex and reveals their activities as immune defense suppressors. Although HopAO1 is predominantly encoded by phylogroup 3 strains isolated from woody organs of woody hosts, both HopAO1 and HopAO2 are phylogenetically clustered according to the woody/herbaceous nature of their host of isolation, suggesting host specialization of the HopAO family across the *P. syringae* complex. HopAO1 and HopAO2 translocate into plant cells and show *hrpL*-dependent expression, which allows their classification as actively deployed type III effectors. Our data also show that HopAO1 and HopAO2 possess phosphatase activity, a hallmark of the members of this family. Both of them exert an inhibitory effect on early plant defense responses, such as ROS production and callose deposition, and are able to suppress ETI responses induced by the effectorless polymutant of *P. syringae* pv. *tomato* DC3000 (DC3000D28E) in *Nicotiana*. Moreover, we demonstrate that a Δ*hopAO1* mutant of *P. savastanoi* NCPBB 3335 exhibits a reduced fitness and virulence in olive plants, which supports the relevance of this effector during the interaction of this strain with its host plants. This work contributes to the field with the first report regarding functional analysis of HopAO homologs encoded by *P. syringae* or *P. savastanoi* strains isolated from woody hosts.

## Introduction

*Pseudomonas syringae* type III secretion system effectors (T3E) are essential elements of the interaction of this bacterial complex with their plant hosts. The study of these effectors has provided a valuable knowledge about the plant immune system, which is the major target of their functions (Deslandes and Rivas, 2012; Dou and Zhou, 2012; Macho, 2016). Immune defenses include the so-called pathogen-associated molecular pattern (PAMP)-triggered immunity (PTI), incited upon pathogen detection, and effector-triggered immunity (ETI), which is initiated by either the direct recognition of effectors or the detection of their effects in the plant cell (Chisholm et al., 2006; Jones and Dangl, 2006; Win et al., 2012). These immune barriers overlap, and defense responses such as callose deposition at the infection site and the production of reactive oxygen species (ROS) have been associated with both of them (Jones and Dangl, 2006; Thomma et al., 2011). The ETI response also involves a specific process that entails the arrest of pathogen progression, the hypersensitive response (HR), which is a localized reaction characterized by the induction of programmed cell death (PCD) (Mur et al., 2008). During a compatible interaction, both layers of defense can be overcome by virulence factors, including T3Es. Thus, these effectors target components of PTI, ETI or both (Boller and Felix, 2009; Katagiri and Tsuda, 2010; Büttner, 2016).

Based on analyses carried out in several phylogenetically diverse model strains of the plant pathogenic bacteria *P. syringae*, the T3E repertoire can be defined by a pool of core effectors and a larger set of variable effectors. These large repertoires of effector proteins interplay and display redundant actions to robustly subvert immunity (Lindeberg et al., 2012; Rafiqi et al., 2012; Shames and Finlay, 2012; Wei et al., 2015). In this complex scene, pathosystems evolve on the basis of PTI-ETI-T3E interactions, which drive the diversification of both defense elements and T3E complexities (Jones and Dangl, 2006; Wei et al., 2015).

Most of the recent progress in the study of T3E has been carried out in *P. syringae* pathosystems involving herbaceous plants. However, knowledge about the infection of woody plants by strains belonging to the genus *Pseudomonas* lags far behind. Although common features with their herbaceous relatives might be found, it should be taken into account that the T3E repertoire of bacterial pathogens isolated from woody hosts, and their functions, might be conditioned by the specific characteristics of woody hosts (Rodriguez-Palenzuela et al., 2010; Matas et al., 2014; Castaneda-Ojeda et al., 2016; Nowell et al., 2016).

The T3E repertoire of *P. savastanoi* pv. *savastanoi* NCPPB 3335, a model pathogen for exploring the bacterial infection of woody hosts, includes 33 T3E, nine of which have been shown to translocate into the plant cell (Rodriguez-Palenzuela et al., 2010; Bardaji et al., 2011; Ramos et al., 2012; Matas et al., 2014; Castaneda-Ojeda et al., 2016). The latest contributions to the study of the function of these effectors have revealed new data concerning the specific role of some of them in interfering with plant defense responses (Matas et al., 2014; Castaneda-Ojeda et al., 2016). Among these T3E candidates, only three are homologs of *P. syringae* pv. *tomato* DC3000. T3E with a previously demonstrated enzymatic function; i.e., HopAO1 (formerly known as HopPtoD2), with tyrosine phosphatase activity (Underwood et al., 2007), HopAB1, with E3 ubiquitin ligase activity (Janjusevic et al., 2006), and HopAF1, with deamidase activity (Washington et al., 2016).

Research on HopAO1 function has revealed its relevant contribution to the virulence of *P. syringae* pv. *tomato* DC3000 in *Arabidopsis* (Bretz et al., 2003), its ability to suppress the HR elicited by an avirulent *P. syringae* strain on *Nicotiana benthamiana* (Espinosa et al., 2003), and in the suppression of the innate immunity induced by the PAMP flagellin in *Arabidopsis* (Underwood et al., 2007). Recent studies have shown that HopAO1 targets elongation factor Tu (EF-Tu) receptor EFR, interfering with the initiation of the immune response after pathogen recognition (Macho et al., 2014), and inhibits proteasome activity in *N. benthamiana* (Üstün et al., 2016). The phylogenetic distribution of effector families in the *P. syringae* effector super-repertoire shows that the T3Es of the HopAO family are distributed among strains belonging to multi-locus sequence typing (MLST) groups I and III established by Baltrus et al. (2011) and Lindeberg et al. (2012). A recent analysis of the distribution of T3Es across phylogroups (PG) 1, 2, and 3 of the *P. syringae* species complex, revealed that PG3 strains *P. savastanoi* pv. *savastanoi* NCPBB 3335, *P. savastanoi* pv. *nerii* ICMP 16943 and *P. savastanoi* pv. *fraxini* ICMP 7711, encode two members of the HopAO family, HopAO1 and HopAO2. Furthermore, these authors showed that HopAO1 is significantly associated with woody hosts across the complex (Nowell et al., 2016). However, translocation of HopAO2 homologs into plant cells has not been demonstrated to date. On the other hand, no functional studies involving HopAO homologs encoded by *P. syringae* or *P. savastanoi* strains isolated from woody host have yet been reported.

In this study, we extended the analysis of the distribution of HopAO1 and HopAO2 to all phylogroups of the *P. syringae* species complex. Our results reveal that the *hopAO1* and *hopAO2* genes are widely distributed in *P. savastanoi* pv. *savastanoi* strains, suggesting a relevant function of these T3E genes during interaction with olive plants. We report the translocation into plant cells and the *hrpL*-dependent expression of these two members of the effector repertoire of the olive pathogen *P. savastanoi* pv. *savastanoi* NCPPB 3335. Our data also show that HopAO1 and HopAO2 possess phosphatase activity, a hallmark of the members of this family, and that they exert an inhibitory effect over defense responses associated with both the PTI and ETI pathways. Moreover, we demonstrate that a Δ*hopAO1* mutant of NCPBB 3335 exhibits reduced fitness and virulence in olive plants, which supports the relevance of this effector during the interaction of *P. savastanoi* NCPBB 3335 with its host plant.

## Materials and Methods

### Distribution of the HopAO1 and HopAO2 across the *P. syringae* Complex

Genome sequences of 100 strains belonging to the *P. syringae* complex were downloaded from GenBank and the presence of HopAO1 and HopAO2 was analyzed by blastn using Geneious software v7.1.13^1^ and the corresponding amino acid sequences of *P. savastanoi* pv. *savastanoi* NCPPB 3335 as template. Positive hits were determined using the “Grade” value, a percentage calculated by Geneious combining the query coverage, *e*-value and identity values for each hit with weights 0.5, 0.25, and 0.25, respectively. Genomes yielding a Grade value ≥92% were considered to encode the corresponding protein.

### Maximum Likelihood Phylogenies

**FIGURE 1:**
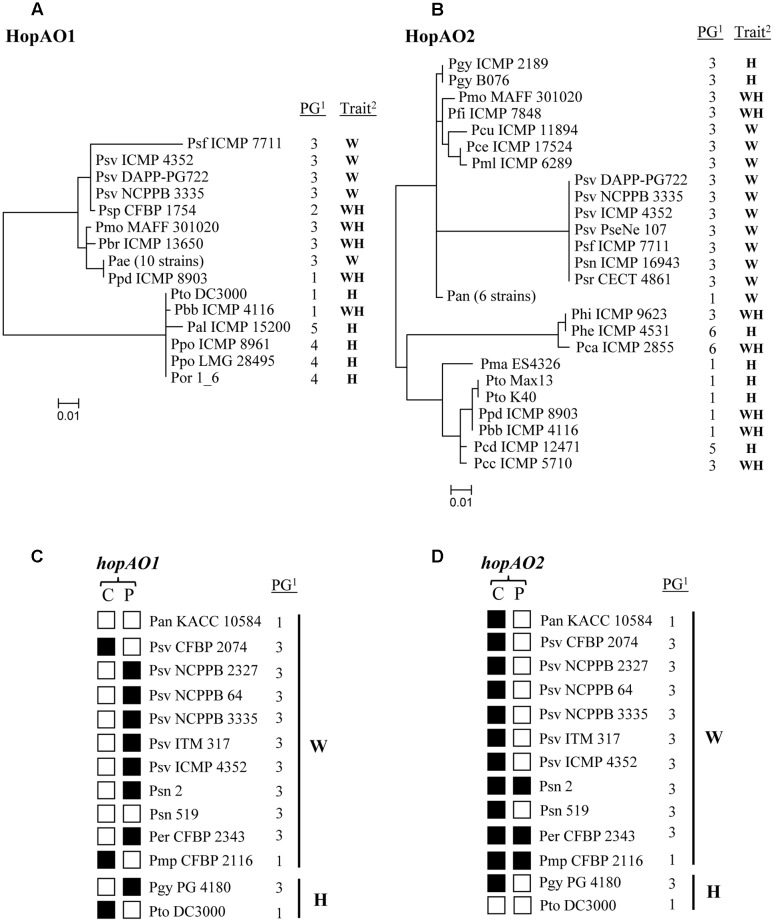
**Distribution and phylogeny of the HopAO family in the *Pseudomonas syringae* complex.** Unrooted maximum likelihood tree of the HopAO1 **(A)** and HopAO2 **(B)** proteins from strains of the *P. syringae* complex. **(C,D)** Distribution of the *P. savastanoi* pv. *savastanoi* NCPPB 3335 T3E genes *hopAO1* and *hopAO2* among a collection of strains of the *P. syringae* complex isolated from woody and herbaceous plant hosts. A dot-blot analysis was performed using the indicated probe. The strains are indicated by their pathovar abbreviation (Supplementary Table S2). Black and white squares represent the presence or absence, respectively, of strong hybridization signals with the indicated effectors for each strain analyzed. ^1^PG, phylogroup (Berge et al., 2014); ^2^Trait: W, woody organ (trunk, stem and/or branches) of woody host; H, herbaceous host; WH, herbaceous organ (leaf) of woody host; C, chromosomal DNA; P, plasmid DNA.

The evolutionary history of HopAO1 and HopAO2 were inferred by using the maximum likelihood method (Jones et al., 1992). The trees with the highest log likelihood (-1584,3692), which are drawn to scale, with branch lengths measured in the number of substitutions per site, are shown (**Figures 1A,B**). All positions containing gaps and missing data were eliminated. Evolutionary analyses were conducted in MEGA7 (Kumar et al., 2016). The accession number of the protein sequences used for the construction of the phylogenetic tree and the abbreviated names used for pathovar designations are presented in Supplementary Table S1.

### Bacterial Strains, Plasmids, Primers, Culture Media, and Growth Conditions

Bacterial strains and plasmids used are described in Supplementary Tables S2, S3, respectively. Primers used in this study for various purposes are described in Supplementary Table S4. All *Pseudomonas* strains were grown in King’s B (KB) medium (King et al., 1954), Lysogeny Broth (LB) medium (Bertani, 1951), Hrp-inducing medium (HIM) (Huynh et al., 1989) or Super Optimal Broth (SOB) (Hanahan, 1983) at 28°C. *Escherichia coli* and *Pseudomonas* strains were grown in LB medium at 37°C or 28°C, respectively. When required, the medium was supplemented with ampicillin (Ap), 100 μg/mL; gentamicin (Gm), 10 μg/mL; kanamycin (Km), 10 or 50 μg/mL; tetracycline (Tc), 10 μg/mL; or spectinomycin (Sp), 10 μg/mL.

Construction of the Δ*hopAO1* (AER-0000610) mutant from *P. savastanoi* pv. *savastanoi* NCPPB 3335 was performed by marker exchange mutagenesis as previously described (Matas et al., 2014), and the correct exchange of the *hopAO1* gene by the Km resistance cassette was determined by Southern blot analysis.

### General DNA Manipulation

Basic DNA and molecular techniques were performed following standard methods (Sambrook and Russell, 2001). Genomic DNA was extracted using the Jet Flex Extraction Kit (Genomed; Löhne, Germany). Plasmid DNA for cloning purposes was extracted using GenElute^TM^ Plasmid Miniprep Kit (Sigma–Aldrich, USA).

### Translocation Assays

The translocation assays were performed as described by Matas et al. (2014). This method is based on the construction of fusion proteins between identified T3E and the calmodulin-dependent Cya reporter domain. Electrocompetent *P. savastanoi* pv. *savastanoi* NCPPB 3335 and NCPPB 3335- T3 cells were transformated with Cya fusions. Sp^R^ transformants were tested by PCR using a forward primer designed specifically and a reverse primer annealing to the cya gene (Supplementary Table S4). Cya assays were performed in *N. tabacum* var. Newdel plants, as previously described (Schechter et al., 2004). *P. savastanoi* pv. *savastanoi* NCPPB 3335 and NCPPB 3335-T3 transformants carrying plasmids expressing T3E-Cya fusions were scraped off of the LB plates, washed twice and resuspended to an optical density at 600 nm (OD_600_) of 0.5 (approximately 10^8^ CFU/mL) in 5 mM morpholinoethanesulfonic acid (pH 5.5) and 100 μM isopropyl β-D-1-thiogalactopyranoside (IPTG). Cell suspensions were injected into the fully expanded upper plant leaves using a 1 mL syringe. Leaf disks were collected at 6 h post-inoculation (hpi) with a 10 mm inner-diameter cork borer, frozen in liquid nitrogen, ground to a powder and suspended in 250 μl of 0.1 M HCl. The samples were incubated at -20°C overnight, and cyclic AMP (cAMP) levels were determined using a 1:100 dilution of the samples and the Correlate-EIA cAMP immunoassay kit according to the manufacturer’s directions (Assay Designs, Inc., Ann Arbor, MI, USA).

The Cya activity of the Cya fusion protein expressed in *E. coli* XL1Blue from pCPP3234 derivatives were assayed as previously reported (Schechter et al., 2004). Bacterial cells were grown in 5 mL of LB medium containing 100 μM IPTG to an OD_600_ of 0.6 to 0.8. The culture was centrifuged, and the pellet was washed and resuspended in sonication buffer (20 mM tris-HCl [pH 8.0] and 10 mM MgCl2). The bacteria were sonicated with a microtip for 2 min, and the cellular debris was pelleted by centrifugation. Cya activity was determined in the presence or absence of bovine calmodulin (Calbiochem, Farmstadt, Germany) by using 5 μl of each lysate (Sory and Cornelis, 1994).

### RNA Extraction and Quantitative RT-PCR Assays

Pure cultures of the wild-type *P. savastanoi* pv. *savastanoi* NCPPB 3335 and its *DhrpL* mutant were grown overnight in KB medium at 28°C. The cells were diluted in fresh KB medium and incubated with shaking at 28°C to an OD_600_ of 0.5. The sample was split into two. One half was pelleted, and frozen (for non-inducing condition) and the other half was pelleted, washed twice with 10 mM MgCl2 and resuspended in the same volume of HIM (Huynh et al., 1989) supplemented with 5 mM mannitol and 0.0006% ferric citrate. After 6 h of incubation, the cells were pelleted and processed for RNA isolation using TriPure Isolation Reagent (Roche Applied Science, Mannheim, Germany) according to the manufacturer’s instructions, except that the TriPure was preheated at 65°C, the lysis step was performed at 65°C, and 1–bromo–3–chloropropane (BCP; Molecular Research Center, Cincinnati, OH, USA) was used instead of chloroform. Total RNA was treated with the RNAeasy kit (QIAGEN) as detailed by the manufacturer. The RNA concentration was determined spectrophotometrically, and its integrity was assessed by agarose gel electrophoresis. DNA-free total RNA was retrotranscribed to cDNA using the cDNA Reverse Transcription kit (Applied Biosystems, Foster City, CA, USA) and random hexamers. The primer efficiency tests, quantitative real-time PCRs (qRT-PCRs) and confirmation of the specificity of the amplification reactions were performed. The relative transcript abundance was calculated using the ΔΔ cycle-threshold (Ct) method (Livak and Schmittgen, 2001). Transcriptional data were normalized to the housekeeping gene gyrA using the Roche LightCycler 480 Software and are presented as the fold change in expression compared to the expression of each gene in the wild-type strain. The relative expression ratio was calculated as the difference in quantitative PCR threshold cycles (ΔCt = Ctgene of interest-CtgyrA). One PCR cycle represents a twofold difference in template abundance; therefore, fold-change values were calculated as 2-ΔΔCt as previously described (Pfaffl, 2001; Rotenberg et al., 2006). qRT-PCRs were performed in triplicate.

### Phosphatase Assays

His-tagged *P. savastanoi* pv. *savastanoi* NCPPB 3335 HopAO1 and HopAO2 were generated using plasmids pENTR-*hopAO1* and pDEST42 as entry and destination vectors, respectively. Site-directed mutagenesis was carried out on pENTR-*hopAO1* using the QuickChange II Site-Directed Mutagenesis Kit (Stratagene, Santa Clara, CA, USA) following the supplier’s instructions. *E. coli* BL21 (DE3) cultures containing plasmids expressing wild-type or both mutant proteins, HopAO1 or HopAO2, were grown at 24°C to an OD_600_ of 0.5 and induced with 0.4 mM IPTG for 4 h Cells were harvested by centrifugation at 5000 g for 15 min, and the pellets were frozen overnight at -20°C. Native and mutant HopAO1 and HopAO2 proteins were purified according to protocol 12 of the QIAexpressionist handbook (Qiagen). The same protocol was used for protein extraction from *E. coli* BL21 (DE3), and the resulting fraction was used as the negative control.

Phosphatase activity was performed using the sensolyte fluorescein diphosphate (FDP) protein phosphatase assay kit (AnaSpec, Fremont, CA, USA) according to the manufacturer’s instructions. This kit provides a fluorogenic assay for measuring the activity of protein phosphatases that convert the fluorescein diphosphate, tetraammonium salt (FDP) into fluorescein, which has a high extinction coefficient and emission quantum yield, thus providing high assay sensitivity. The phosphatase assay was performed by mixing 50 μl of a protein containing sample with 50 μl of FDP reaction solution. The reaction was incubated at 25°C for 30 min, and 50 μl of stop solution was added to stop the reaction. The fluorescence signal was measured using excitation/emission = 485 nm/535 nm. As a negative control, samples without protein (distilled water) were used. The phosphatase activity for the control sublines was set at 100%, and the respective values for the experiment subline were calculated with respect to that value.

### Plant Bioassays

The *N. benthamiana* and *N. tabacum* (var. Newdel) plants used in this study were 5–7 and 4–6 weeks old, respectively. The plants were grown with a 16-h light/8-h dark photoperiod with day/night temperatures of 26°C/22°C. *N. tabacum* var. Newdel plants were used for the HR assays. The leaves were infiltrated with bacterial suspensions (10^7^ and 10^8^ CFU/mL) of *P. syringae pv. tomato* DC3000D28E or its derivatives harboring plasmids expressing each of the different *P. savastanoi pv. savastanoi* T3E (Supplementary Table S3). The generated symptoms, scored 48 h after inoculation, were captured with a high-resolution digital camera (Nikon DXM 1200, Nikon Corporation, Tokyo, Japan).

For competition assays, the *N. benthamiana* leaves were inoculated with mixed suspensions containing equal CFU (approximately 10^4^ CFU/mL) of *P. syringae* pv. *tomato* DC3000D28E and each of its transformants carrying pCPP5040 derivatives expressing *P. savastanoi* pv. *savastanoi* T3E (Supplementary Table S3). Input and output pools, assayed 1 and 6 h after inoculation, respectively, were plated onto LB-Sp and LB-Sp-Gm to select for DC3000D28E and the transformants with pCPP5040 derivatives, respectively. A competitive index (CI) was calculated by dividing the output ratio (CFU transformant: CFU DC3000D28E) by the input ratio (CFU transformant: CFU DC3000D28E). Competition indices of the transformant strains expressing *P. savastanoi* pv. *savastanoi* T3E versus DC3000D28E were normalized with respect to the CI obtained for DC3000D28E (pCPP5040). The CIs presented represent the mean of three replicates demonstrating typical results from three independent experiments. The results were statistically analyzed using Student’s *t*-test.

Olive plants derived from seed germinated *in vitro* (originally collected from an ‘Arbequina’ plant) were micropropagated, rooted, and maintained as previously described (Rodríguez-Moreno et al., 2008, 2009). To analyze the pathogenicity of the *P. savastanoi* pv. *savastanoi* NCPPB 3335 *hopAO1* mutant in 1-year-old olive explants (woody plants), micropropagated olive plants were transferred into soil and maintained in a glasshouse at 27°C with a relative humidity of 58% under natural daylight. The plant stems were wounded and infected with approximately 10^6^ CFU of *P. savastanoi* pv. *savastanoi* strains. Morphological changes scored at 28 (in young explants) or at 90 days (in 1-year explants) post-inoculation, were captured with a high-resolution digital camera (Nikon DXM 1200).

### Detection of ROS Production and Callose Deposition

Reactive oxygen species production was observed after 3-3′-diaminobenzidine (DAB) staining (Thordal-Christensen et al., 1997) 4 h after inoculation with *P. fluorescens* Pf55 [pLN18] derivatives. Bacterial suspensions (10^8^ CFU/mL) were inoculated into *N. tabacum* var. Xanthi leaves using a blunt syringe. Small pieces of tobacco leaves cut from around the injection area were placed into a syringe and stained by vacuum infiltration of a freshly prepared 1 mg/mL solution of DAB (Sigma–Aldrich D-8001, Sigma–Aldrich, Inc., St. Louis, MO, USA) in 8 mM HCl, pH 3.8. Chlorophyll was removed by submerging the leaves into a solution of ethanol/lactic acid/glycerol [3:1:1 (vol/vol/vol)] at 60°C and stored overnight at room temperature on water-soaked filter paper. At least ten biological replicates from each specimen were mounted on slides in a 50% glycerol (vol/vol) solution and observed with a Nikon Eclipse E800 microscope (Nikon Corporation, Tokyo, Japan) under bright field. DAB staining produces an intensely brown precipitate at the sites of ROS production, which were next to the infection zone.

Callose deposition samples were developed 12 h after inoculation and stained as previously described (Guo et al., 2009). Chlorophyll was removed in 95% (vol/vol) ethanol from small pieces of tobacco leaves, which were cut from around the injection area, and staining was performed in a 0.02% (wt/vol) solution of aniline blue (Sigma–Aldrich #415049, Sigma–Aldrich, Inc., St. Louis, MO, USA) in 150 mM potassium phosphate, pH 9, for 1 h in the dark. At least ten biological replicates from each specimen were mounted in 50% (vol/vol) glycerol on glass slides. Observations were conducted under UV-light excitation using the filter UV-2^a^ (EX 330- 380, DM 400; BA 420) on a Nikon Eclipse E 800 microscope (Nikon Corporation, Tokyo, Japan).

Reactive oxygen species production and callose deposition were quantified as previously described (Rodríguez-Herva et al., 2012) with slight modifications. Up to four snapshots of each specimen from equivalent areas surrounding the wound (inoculation zone) were captured with a Nikon DXM1200 camera using the Nikon ACT-1 2.70 software. The same settings and a final magnification of 40X were applied to all the samples. After calibrating all the images using the scale bar included in each picture, DAB staining and aniline blue fluorescence were quantified using the program Visilog 6.3 (Noesis, Les Ulis, France). For this purpose, the characteristic brown color of the DAB precipitate and the specific blue fluorescence of callose deposition were separated by color deconvolution using the i_classification command. Then, the stained areas were quantified, and the results were expressed in mm^2^. Five-six images per assay were analyzed, and statistical analyses were performed using one-way ANOVA followed by *post hoc* comparisons using Tukey test.

## Results

### Identification and Distribution of HopAO1 and HopAO2 among *P. savastanoi* pv. *savastanoi* and Related Strains

Prediction of T3E genes in the draft genome sequence of *P. savastanoi* pv. *savastanoi* NCPPB 3335 previously allowed the identification of a HopAO1 homolog located in plasmid pPsv48B (Rodriguez-Palenzuela et al., 2010; Bardaji et al., 2011). A chromosomally encoded candidate HopAO2 T3E is also found in NCPPB 3335 (accession number GG774693.1, locus tag PSA3335_5047), which shows 93% amino acid identity (100% cover) with its corresponding *P. syringae* pv. *actinidiae* MAFF302091 homolog and only 33% identity with HopAO1 from NCPPB 3335 (**Supplementary Figure S1**). Thus, NCPBB 3335 encodes two dissimilar T3E candidates of the HopAO family (HopAO1 and HopAO2). Proteins belonging to this family share the signature sequence [LIVMF]HCxAGxxR[STC][STAG], characteristic of the active site of the protein tyrosine phosphatase (PTP) family (Fauman and Saper, 1996). Further analyses of the amino acid sequences of NCPPB 3335 HopAO1 and HopAO2 showed that, in fact, both of them harbor this conserved motif.

With the aim of extending the analysis of the prevalence of HopAO1 and HopAO2 homologs to all established phylogroups of the *P. syringae* complex (Berge et al., 2014; Baltrus et al., 2017), a total of 100 genomes corresponding to all *Pseudomonas* species and pathovars of the *P. syringae* complex which genomes have been sequenced were downloaded from GenBank and the presence of the HopAO1 and HopAO2 was analyzed using Geneious software v7.1.13^2^. This study was facilitated by the recent publication of the draft genome sequences of numerous strains of the *P. syringae* species complex (Moretti et al., 2014; Bartoli et al., 2015; Nowell et al., 2016; Thakur et al., 2016). The HopAO1 phylogeny was reconstructed from the 462-468 amino acids alignment of 24 orthologous using maximum likelihood (**Figure 1A**). The HopAO1 sequences of *P. savastanoi* pv. *savastanoi* PseNe 107, *P. savastanoi* pv. *nerii* ICMP 16943 and *P. savastanoi* pv. *retacarpa* CECT 4861, which are possibly truncated, were not included in this analysis. The results revealed the partitioning of these strains into two main clusters, one of them predominantly including PG3 strains isolated from woody hosts. This cluster also includes PG1 strain *P. syringae* pv. *philadelphi* ICMP 8903 and PG2 strain *P. syringae* pv. *papulans* CFBP 1754, isolated from leaf spots of mock orange and apple, respectively. While ICMP 8903 is included in the “aesculi clade,” comprising all 10 *P. syringae* pv. *aesculi* strains analyzed, CFBP 1754 clusters together with *P. savastanoi* pv. *fraxini* ICMP 7711 (isolated from ash) and all three *P. savastanoi* pv. *savastanoi* strains analyzed, i.e., NCPPB 3335, DAPP-PG722 and ICMP 4352. The second cluster is composed by six strains, five of which were isolated from herbaceous hosts, i.e., *P. syringae* pv. *tomato* DC3000 (PG1), PG4 strains *P. syringae* pv. *oryzae* 1-6 and *P. syringae* pv. *porri* ICMP 8961 and LMG 28495 and, *P. syringae* pv. *alisalensis* ICMP 15200 (PG5). The remaining strain, *P. syringae* pv. *berberidis* ICMP 4116, belongs to PG1 and was isolated from leaf spots of barberry (**Figure 1A**).

The HopAO2 phylogeny was reconstructed from the 338-339 amino acids alignment of 30 orthologous using maximum likelihood (**Figure 1B**). In a similar way than HopAO1, one of these clusters is predominantly composed by PG3 strains isolated from woody organs of woody hosts, with the exception of all six *P. syringae* pv. *actinidiae* (PG1) strains analyzed, isolated from kiwifruit, and *P. syringae* pv. *glycinea* strains ICMP2189 and B076, also PG3 strains isolated from soybean (**Figure 1B**). The second cluster, which is divided in two main branches, is composed exclusively by 10 strains isolated from either herbaceous hosts or leaf spots of woody hosts. Although all five PG1 strains cluster together in the same sub-branch, i.e., *P. syringae* pv. *berberidis* ICMP 4116, *P. syringae* pv. *maculicola* ES4326, *P. syringae* pv. *philadelphi* ICMP 8903 and *P. syringae* pv. *tomato* Max13 and K40, the distribution of the remaining strains is not related to their PG.

The *hopAO1* and *hopAO2 P. savastanoi* pv. *savastanoi* NCPPB 3335 T3E genes were used as probes in dot-blot hybridizations to ascertain their distribution among a collection of 31 *P. savastanoi* pv. *savastanoi* strains isolated in different countries and among a selection of *P. syringae* strains isolated from either woody or herbaceous hosts (Supplementary Table S2). The probes, which were specific for each of the *hopAO* alleles, were obtained by PCR using primer pairs *hopAO1*-F/*hopAO1*-R (*hopAO1*) and *hopAO2*-F/*hopAO2*-R (*hopAO2*) (Supplementary Table S4). The results showed that 30 out of the 31 *P. savastanoi* pv. *savastanoi* strains, as well as *P. savastanoi* pv. *nerii* 2, *P. syringae* pv. *morsprunorum* CFBP 2116, *P. syringae* pv. *eriobotryae* CFBP 2343 and *P. syringae* pv. *glycinea* PG4180, hybridized with both probes (data not shown). Conversely, strains belonging to *P. syringae* pathovars *phaseolicola*, *syringae*, *lachrymans*, *dendropanacis*, *sesami*, and *alisalensis* did not hybridize with any of these probes (data not shown) and were discarded for further analysis. Thus, with the exception of *P. syringae* pv. *glycinea* PG4180 (PG3, isolated from an herbaceous host) and *P. syringae* pv. *morsprunorum* CFBP 2116 (PG1, isolated from a woody host), all other strains encoding both HopAO1 and HopAO2 sequences were isolated from woody hosts and belong to PG3 (**Figure 1**).

Plasmid preparations and total DNA isolated from a selection of six *P. savastanoi* pv. *savastanoi* strains hybridizing with both *hopAO1* and *hopAO2* probes in the dot-blot analysis, as well as from all other strains hybridizing with at least one of the two probes, were analyzed by southern-blot hybridization to determine the localization of these genes on their replicons. While *hopAO1* was found to be plasmid-encoded in five out of the six *P. savastanoi* pv. *savastanoi* strains used, *hopAO2* was detected in the chromosome in the six strains (**Figures 1C,D**).

### HopAO1 and HopAO2 Are Translocated via the *P. savastanoi* pv. *savastanoi* T3SS

**FIGURE 2:**
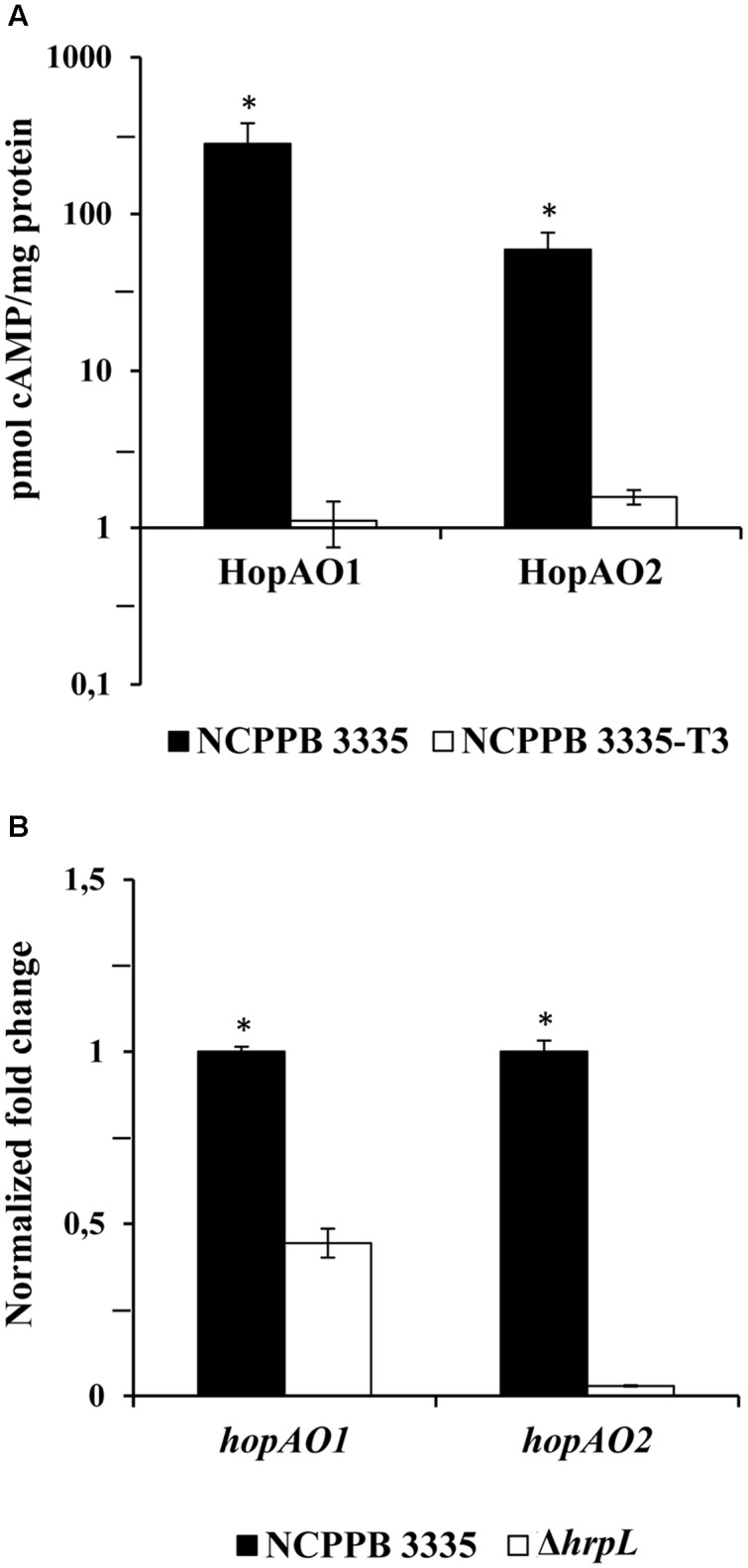
**Validation of HopAO1 and HopAO2 as members of the *P. savastanoi* pv. *savastanoi* NCPPB 3335 T3SS effectors repertoire.** **(A)** Translocation assay of HopAO1 and HopAO2. CyaA dependent production of cAMP was used to measure the translocation of the T3E-Cya fusions into the *N. tabacum* cv. Newdel plants. The plants were inoculated with *P. savastanoi* pv. *savastanoi* NCPPB 3335 or NCPPB 3335-T3 (the T3SS mutant) expressing the indicated Hop-Cya fusions from pCPP3234 derivatives. The values represent the mean and standard error for the samples obtained in triplicate; similar results were obtained in multiple experiments. The asterisks indicate significant differences (*P* = 0.05) between the cAMP levels obtained for the NCPPB 3335 and NCPPB 3335-T3 strains. **(B)** HrpL-dependent expression of the *hopAO1* and *hopAO2* genes. qRT-PCR with the indicated T3E genes in NCPPB 3335 versus NCPPB 3335 Δ*hrpL* 6 h after transferring to the Hrp-inducing medium (HIM). The fold change was calculated after normalization using the *gyrA* gene as an internal control. The results represent the means from three independent experiments. The error bars represent the standard deviation. The asterisks indicate significant differences (*P* = 0.05) between the expression values obtained for NCPPB 3335 and the Δ*hrpL* mutant.

To determine whether the selected candidate effectors were T3SS substrates that could be translocated into plant cells, pCPP3234 derivatives expressing fusions of *Bordetella pertussis* Cya to the C terminus of full-length HopAO1 and HopAO2 were constructed. This system, which is based on cAMP production exclusively in the presence of eukaryotic calmodulin, has been widely used for analyzing the translocation of *P. syringae* T3E (Sory and Cornelis, 1994; Casper-Lindley et al., 2002; Schechter et al., 2004). *N. tabacum* leaves were infiltrated with either *P. savastanoi* pv. *savastanoi* NCPPB 3335 or the strain NCPPB 3335-T3, a T3SS mutant derived from wild-type NCPPB 3335 (Pérez-Martínez et al., 2010), expressing each of the two constructed Cya fusions. As illustrated in **Figure 2A**, significant differences in cAMP production between the wild-type strain and the T3SS mutant strain were observed for both T3E candidates tested—HopAO1, HopAO2—indicative of their translocation through *P. savastanoi* pv. *savastanoi* T3SS.

An identifiable Hrp box (Fouts et al., 2002) was found in the 100 nucleotides upstream of the start codon of both *hopAO1* and *hopAO2* candidates from *P. savastanoi* pv. *savastanoi* NCPPB 3335 (Supplementary Table S5). To unveil the HrpL-dependent expression of *hopAO1* and *hopAO2* in *P. savastanoi* pv. *savastanoi* NCPPB 3335, the expression of *hopAO1* and *hopAO2* was analyzed using qRT-PCR in both the wild-type and the NCPPB 3335 Δ*hrpL* mutant. Under inducing conditions (cells grown 6 h in HIM), the expression of the *hopAO1* and *hopAO2* genes decreased (0.4- and 0.02-fold, respectively) in the *ΔhrpL* mutant compared to the wild-type, indicating an expression dependency on HrpL (**Figure 2B**).

### HopAO1 and HopAO2 Possess Phosphatase Activity

**FIGURE 3:**
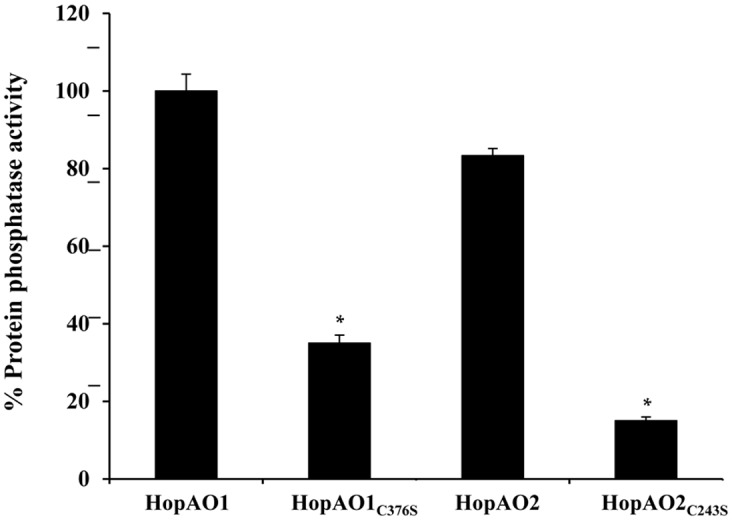
**Phosphatase catalytic activity of *P. savastanoi* pv. *savastanoi* HopAO1 and HopAO2.** Phosphatase activity was assayed using FDP. Values were normalized to the phosphatase activity obtained for an empty vector (negative control). The results represent the means from three independent reactions. Asterisks indicate values significantly different between the phosphatase activity obtained for the wild-type HopAO1 or HopAO2 protein and their protein mutants, respectively. Statistical analyses were performed using Student’s *t*-test with a threshold of *P* = 0.05.

The HopAO T3E family is characterized on the basis of a conserved PTP domain (Bretz et al., 2003; Espinosa et al., 2003), with activity dependent on a cysteine located at position 378 of HopAO1 from *P. syringae* pv. *tomato* DC3000 (Bretz et al., 2003). To determine whether *P. savastanoi* pv. *savastanoi* NCPPB 3335 HopAO1 and HopAO2 are also active protein phosphatases, affinity-purified HopAO1-His_6_ and HopAO2-His_6_ were used in a phosphatase activity *in vitro* assay performed under non-denaturing conditions. We also tested the activity of site-directed HopAO1 and HopAO2 mutants involving the substitution of the PTP-related Cys by a Ser, HopAO1_C376S_ and HopAO2_C243S_, respectively. FDP hydrolysis was clearly detected for wild-type HopAO1-His_6_ and HopAO2-His_6_; however, the percentage of hydrolysed FDP significantly decreased in both mutant proteins (**Figure 3**). The results demonstrate that the phosphatase activity shown *in vitro* by *P. savastanoi* pv. *savastanoi* NCPPB 3335 HopAO1 and HopAO2 is dependent on its Cys_376_ or Cys_243_ amino acid residue, respectively.

### *P. savastanoi* pv. *savastanoi* NCPPB 3335 T3SS Effectors HopAO1 and HopAO2 Inhibit Early Plant Defense Responses in *N. tabacum*

**FIGURE 4:**
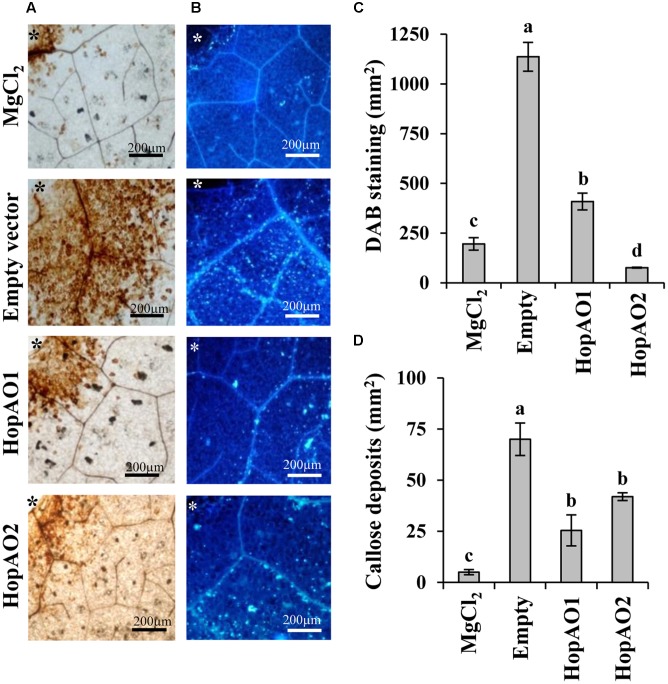
**Interference with plant immune responses by the *P. savastanoi* pv. *savastanoi* T3SS effectors HopAO1 and HopAO2.** **(A,B)** DAB staining and callose deposition in the *N. tabacum* var. Xhanti leaves. The plants were challenged with *P. fluorescens* 55 [pLN18] harboring the pCPP5040 empty vector or the vectors expressing *P. savastanoi* pv. *savastanoi* NCPPB 3335 T3SS effectors. The DAB signal was quantified 4 h after inoculation **(A)**, and the callose deposition was detected by aniline blue staining and quantified 12 h after infection **(B)**. **(C,D)** Quantification of the DAB staining (ROS production) and callose deposition, respectively. For the histograms, the data are the means ± standard error of the mean for at least five replicates; the bars topped with the same letter represent the values that are not significantly different using a one-way ANOVA and Tukey’s test for multiple comparisons (*P* = 0.01). Asterisks indicate the inoculation zone.

The suppression of early plant defense responses such as ROS production and callose deposition by HopAO1 and HopAO2 was tested using the heterologous expression system *P. fluorescens* 55 (Pf55) [pLN18] (Jamir et al., 2004; López-Solanilla et al., 2004; Oh et al., 2010; Matas et al., 2014). This system has been previously used to demonstrate the suppression activity of PTI by other *P. savastanoi* NCPPB 3335 T3SS effectors (Matas et al., 2014). *P. savastanoi* pv. *savastanoi* NCPPB 3335 HopAO1 and HopAO2 were expressed in Pf55 [pLN18], which induces a PTI response in inoculated plants while enabling the delivery of effector proteins into plant cells. After challenging *N. tabacum* leaves with the derivative strains, we analyzed ROS production and callose deposition. **Figure 4** shows that the ROS levels determined by DAB staining were significantly reduced by the expression of HopAO1 or HopAO2 compared to the control strain Pf55 [pLN18] harboring an empty vector (Tukey’s test; *P* ≤ 0.01) (**Figures 4A,C**). Moreover, both T3Es significantly reduced the levels of callose deposition compared to the control strain (Tukey’s test; *P* ≤ 0.01) (**Figures 4B,D**). Therefore, HopAO1 and HopAO2 are able to interfere with the early innate immune responses of the plant.

### HopAO1 and HopAO2 Suppress ETI Responses Induced by the Effectorless Polymutant of *P. syringae* pv. *tomato* DC3000

**FIGURE 5:**
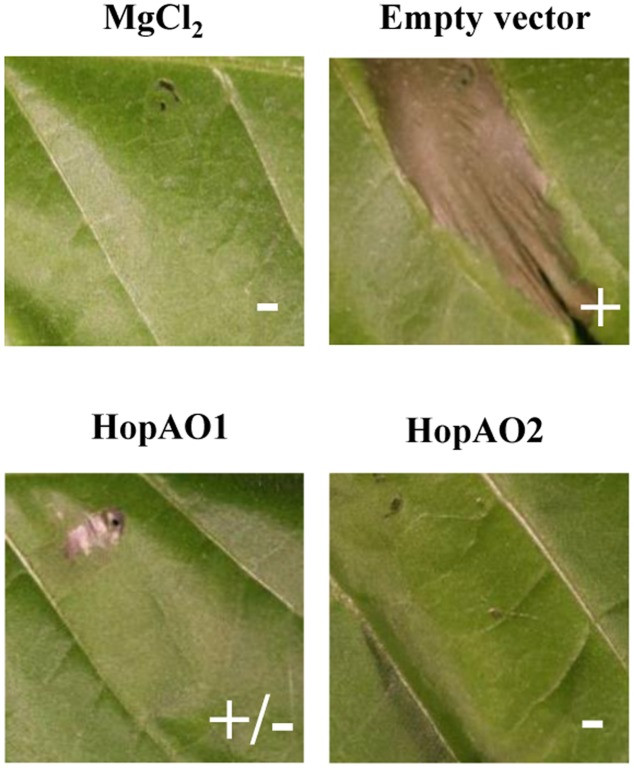
**Interference with *N. tabacum* cell death by the *P. savastanoi* pv. *savastanoi* T3SS effectors HopAO1 and HopAO2.** Cell death response in the *N. tabacum* var. Newdel leaves 48 h after inoculation with *P. syringae* pv. *tomato* DC3000D28E harboring pCPP5040 (empty vector) or derivatives expressing HopAO1 or HopAO2. Cell death response: +, positive; –, negative. The bacterial cells were adjusted to 2 × 10^8^ CFU/mL. Each experiment was repeated at least three times with similar results.

Effector-triggered immunity responses constitute the second layer of immune defenses which overlap with PTI responses (Lindeberg et al., 2012). To determine if *P. savastanoi* pv. *savastanoi* NCPPB 3335 HopAO1 and HopAO2 functions interfere with this layer of defense, we expressed these two proteins in the *P. syringae* pv. *tomato* strain DC3000D28E (Cunnac et al., 2011). This polymutant strain harbors deletions in all 28 well-expressed effector genes and is considered functionally effectorless but otherwise wild-type *in planta.* The HR elicitation in *N. benthamiana* and *N. tabacum* can be explained by the fact that DC3000D28E has the wild-type complement of T3SS helper proteins (except HrpW1), unknown effectors or weakly expressed T3Es, which can elicit plant defenses and induce an HR response (Kvitko et al., 2007; Cunnac et al., 2011). It has been recently demonstrated that the induction of cell death by this strain in *N. benthamiana* is solely dependent on HopAD1 (Wei et al., 2015). We analyzed the ability of the DC3000D28E derivatives expressing HopAO1 or HopAO2 to elicit cell death in *N. tabacum* compared to the DC3000D28E strain. After 48 h of treatment, the polymutant strain stimulated an ETI-like response that was partially and completely inhibited by the expression of the *P. savastanoi* pv. *savastanoi* NCPPB 3335 proteins HopAO1 and HopAO2, respectively (**Figure 5**). These results suggest that these two effectors participate in the inhibition of the plant defense response associated with the onset of PCD.

DC3000D28E has been shown to grow better *in planta* when coinoculated with a strain that is able to suppress plant immunity, such as DC3000Δ*hopQ1-1* (Cunnac et al., 2011). The ability of HopAO1 and HopAO2 to restore the growth of DC3000D28E *in planta* was tested in *N. benthamiana* leaves. For this purpose, competition assays between the polymutant strain (DC3000D28E) and each derivative expressing either HopAO1 or HopAO2 were conducted. *N. benthamiana* leaves were infiltrated with a mixed inoculum (1:1) of DC3000D28E and each of the derivatives; after 6 days, the bacteria were recovered, and viable cells were determined. Interestingly, the expression of *P. savastanoi* pv. *savastanoi* HopAO1 or HopAO2 in DC3000D28E significantly increased the competitiveness of the strain, which was reflected in a CI value (HopAO1/DC3000D28E or HopAO2/DC3000D28E) as a mean ± standard error of three replicates of 2.14 ± 0.078 and 1.82 ± 0.062, respectively. These values are significantly different from unity (statistical analyses were performed using Student’s *t*-test with a threshold of *P* = 0.05).

### A *P. savastanoi* pv. *savastanoi* NCPPB 3335 *hopAO1* Mutant Is Hypovirulent in Olive Plants

To analyze the impact of the immune suppression described above for HopAO1 and HopAO2 in the virulence of *P. savastanoi* pv. *savastanoi* NCPBB3335, we decided to construct mutant strains affected in each of these two genes. While construction of the Δ*hopAO1* mutant strain by marker exchange was straightforward, the isolation of a Δ*hopAO2* mutant using this technique was unsuccessful. Thus, we decided to approach the inactivation of *hopAO2* by plasmid insertion using an internal fragment of this gene. However, all attempts to insert the plasmid into the NCPPB 3335 *hopAO2* gene also failed, indicating that this gene is located in a genomic region of low recombination.

**FIGURE 6:**
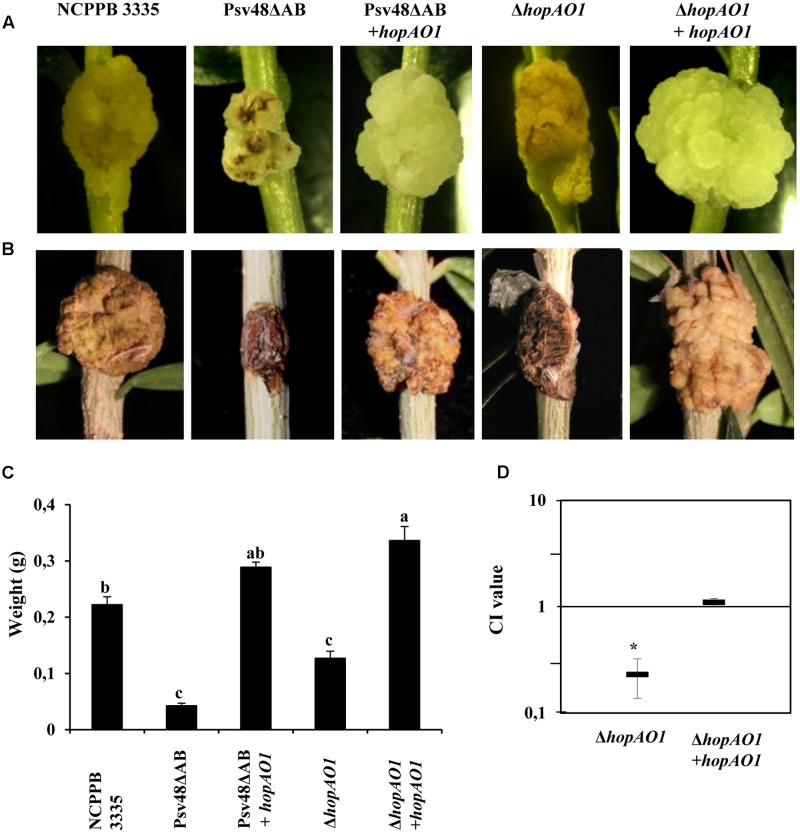
**Role of the HopAO1 gene in the virulence of *P. savastanoi* pv. *savastanoi* NCPPB 3335 in olive plants.** Knots induced by the indicated strains on young micropropagated olive plants at 28 days post-inoculation (dpi; **A**) and in 1-year-old olive plants at 90 dpi **(B)**. **(C)** Weight of knots developed on lignified olive plants infected with the indicated strains. Knot weights are the means of six different knots. Statistical analyses were performed using one-way ANOVA. **(D)** Competitive index for mixed inoculations of NCPPB 3335 and its derivative strains in micropropagated olive plants. The error bars indicate the standard error from the average of three different assays. The asterisks indicate values significantly different from one. Statistical analyses were performed using Student’s *t*-test with a threshold of *P* = 0.05. NCPPB 3335, wild-type strain; Psv48DAB NCPPB 3335 cured of plasmid pPsv48A and pPsv48B; Psv48DAB + *hopAO1*, Psv48DAB expressing *hopAO1*; D*hopAO1*, NCPPB 3335 D*hopAO1* mutant; D*hopAO1* + *hopAO1*, D*hopAO1* expressing *hopAO1*.

It has been previously described that the inoculation of olive plants with a *P. savastanoi* pv. *savastanoi* NCPPB 3335 mutant cured of plasmids pPsv48A and pPsv48B (strain Psv48ΔAB), which encode T3E HopAF1 and HopAO1, respectively, induced attenuated hyperplastic knots, and necrosis (Bardaji et al., 2011). Consequently, complemented Psv48ΔAB and Δ*hopAO1* strains expressing HopAO1 were also constructed and inoculated in both *in vitro* micropropagated and lignified olive plants. In agreement with Bardaji et al. (2011), Psv48ΔAB induced less severe knots than the wild-type strain in both plant systems (**Figures 6A–C**). Interestingly, although the sizes of the knots induced by the Δ*hopAO1* mutant at 28 dpi on the stem of *in vitro-*grown olive plants were not significantly different from those induced by the wild-type strain, they showed necrotic lesions similar to those from plants inoculated with the Psv48ΔAB mutant (**Figure 6A**). In addition, the competitiveness of the Δ*hopAO1* mutant in this plant system was compromised compared with the wild-type strain (**Figure 6D**). Additionally, the weights of the knots induced by the Δ*hopAO1* mutant in lignified olive plants, which also showed an increased necrosis, were significantly lower than those developed by the wild-type strain (**Figure 6C**). The ectopic expression of the *hopAO1* gene in both the Δ*hopAO1* mutant and the plasmid-cured strain Psv48ΔAB fully restored the wild-type appearance of the knots in both plant systems (**Figures 6A,B**). Complementation of the Δ*hopAO1* mutant also resulted in the restoration of the competitiveness of the strain in olive plants (**Figure 6D**). Altogether, these results reveal a significant role of HopAO1 during the infection process in olive plants.

## Discussion

Distribution of effectors among strains with different host ranges is becoming a hallmark of the evolutionary adaptation of bacterial pathogens to their corresponding hosts. We have analyzed the presence among different *P. syringae* strains of two members of the HopAO family (HopAO1 and HopAO2) identified in the pathogen of woody hosts *P. savastanoi* NCPPB 335. Results obtained for HopAO1 are in agreement with the previously reported significant association of HopAO1 with woody hosts (Nowell et al., 2016) and suggest diversification of this T3E across the *P. syringae* complex according to the woody/herbaceous nature of their host of isolation. In the case of HopAO2, the results also showed the partitioning of the strains into two separated clusters according the woody/herbaceous nature of their host of isolation. Furthermore, codification of both HopAO1 and HopAO2 is restricted to only a few PG1 (*P. syringae* pv. *berberidis* ICMP 4116 and *P. syringae* pv. *philadelphi* ICMP 8903) and PG3 (all *P. savastanoi* strains analyzed and *P. syringae mori* MAFF 301020) strains isolated from woody hosts, suggesting a relevant role of the HopAO family during interaction with woody hosts.

The preferential codification of the *hopAO1* gene in a plasmid of strains belonging to phylogroup three isolated from woody hosts (**Figure 1C**), previously reported for *P. savastanoi* pv. *savastanoi* NCPPB 3335 (Bardaji et al., 2011), provides evidence of horizontal transfer among this group of bacteria. On the other hand, the phylogeny of HopAO2 is not congruent with that of the *P. syringae* complex deduced from housekeeping genes (Berge et al., 2014), also suggesting the existence of horizontal transfer (**Figure 1B**). Although neither of these T3Es is restricted to *P. syringae* pathovars infecting woody hosts, both HopAO1 and HopAO2 are phylogenetically clustered according to the woody/herbaceous nature of their host of isolation, suggesting the diversification of the amino acid sequences of these effectors to adapt to their respective hosts. Additionally, the simultaneous occurrence of these two effectors among *P. savastanoi* pv. *savastanoi* strains (**Figure 1C**) suggests that both of them might play an important role during the interaction of this pathogen with olive plants.

The HopAO1 and HopAO2 translocation to plant cells (**Figure 2A**) and their transcriptional dependency on HrpL (**Figure 2B**) demonstrate that *P. savastanoi* pv. *savastanoi* NCPPB 3335 *hopAO1* and *hopAO2* genes encode actively deployed T3E. These *P. savastanoi* effectors present the characteristic signature sequence of the active site of the PTP family (Fauman and Saper, 1996). We have showed that HopAO1 and HopAO2 display phosphatase activity *in vitro*. Tyrosine phosphatase activity has been previously described for the *P. syringae* pv. *tomato* HopAO1 homolog (Espinosa et al., 2003). Moreover, the catalytic activity of this effector is partially responsible for the reduction of the Tyr phosphorylation level of the EFR receptor upon PAMP treatment, inhibiting their ligand-induced activation and downstream immune signaling (Macho et al., 2014). *P. syringae* pv. *tomato* DC3000 HopAO1 has been also recently demonstrated to interact with and inhibit responses associated with the Pattern-Recognition Receptor (PRR) FLS2 (Macho et al., 2014). These membrane-bound receptors (Gómez-Gómez and Boller, 2000; Zipfel et al., 2004, 2006) are important for anti-bacterial immunity and recognize the bacterial PAMPs EF-Tu (elf18) and flagellin (flg22) (Boller and Felix, 2009), respectively. The inhibition of callose deposition and ROS formation in *N. tabacum* (**Figures 4A,B**) by NCPPB 3335 HopAO1 and HopAO2, together with the presented phosphatase activity, is in agreement with the phenotypes reported for the targeting of the host PRRs by DC3000 HopAO1 (Macho et al., 2014). The recognition of PAMPs by PRRs constitutes the initial step of the signaling process involving PTI responses. Furthermore, it has been recently described that proteasome function is required for PTI responses and that HopAO1 acts as an inhibitor of this activity, probably through the reduction of the phosphorylation status of certain proteasome subunits (Üstün et al., 2016).

Considering the induction of PTI as a signaling process in which early and late phases have been described (Lindeberg et al., 2012), it could be hypothesized that the function of these two members could involve the targeting of processes upstream of the response phases and thus at the level of the PAMPs recognition. However, future research unveiling the plant targets of these effectors will allow determining if both of them target the same process associated with the onset of the plant immune response.

Previous studies have shown that the functionally effectorless strain *P. syringae* DC3000D28E is suitable for testing the ability of T3E to restore bacterial growth *in planta* and to modulate plant defense responses (Cunnac et al., 2011). Our results indicate that both HopAO1 and HopAO2 can interfere with the initiation of the ETI-like response induced by DC3000D28E in tobacco plants (**Figure 5**). DC3000D28E strain is able to induce a cell death response both in *N. benthamina* and *N. tabacum* in a dose dependent manner (Cunnac et al., 2011). It could be hypothesized that, as it has been described in *N. benthamiana*, the sole responsible of the cell death elicitation is HopAD1 (Wei et al., 2015). In that case *P. savastanoi* pv. *savastanoi* NCPPB 3335 HopAO1 and HopAO2 proteins would function inhibiting the cell death elicited by this effector. However, it cannot be discarded that other weak T3E belonging to the VIII gene cluster (Wei et al., 2007), or helper proteins as harpins, which are present in this strain, could be also responsible of the cell death elicitation interfered by NCPPB 3335 HopAO1 and HopAO2.

In addition, the expression of either HopAO1 or HopAO2 in DC3000D28E increased the competitiveness of this strain in *N. benthamiana.* The growth restoration of DC3000D28E in *N. benthamiana* by the expression of single T3E in this strain has been reported for only *P. syringae* pv. *tomato* DC3000 AvrPto and AvrPtoB (Cunnac et al., 2011), as well as for *P. savastanoi* pv. *savastanoi* NCPPB 3335 effectors HopBL2 and HopAF1-2 (Matas et al., 2014). These results are in agreement with the observed delay in the onset of HR induced by *P. syringae* pv. *phaseolicola* in the non-host plant *N. tabacum* when expressing *P. syringae* pv. *tomato* DC3000 HopAO1 (Espinosa et al., 2003).

Effector-triggered immunity suppression is now thought to be an ability of many *P. syringae* T3E (Guo et al., 2009). Moreover, effectors belonging to different families have been previously described to have distinct roles in interactions with the host immune system, including the suppression of different PRRs and both the elicitation and suppression of ETI, as is the case for AvrPtoB or VirPphA (Jackson et al., 1999; Abramovitch et al., 2003; Rosebrock et al., 2007; Cheng et al., 2011). Actually, individual T3Es may interact with multiple immunity-associated proteins in plants (Bogdanove and Martin, 2000; Mukhtar et al., 2011; Singh et al., 2014; Weßling et al., 2014). *P. savastanoi* pv. *savastanoi* NCPPB 3335 HopAO1 and HopAO2 effectors could be involved in the interference of both early immunity PTI responses at the level of PRR suppression and ETI downstream pathways or alternatively act at convergence points of many defense pathways. Furthermore, the interplay between effector activities described previously (Wei et al., 2015), which enable pathogen adaptation to its host, might be the mode of action behind the inhibitory effects reported here for NCPPB 3335 HopAO1 and HopAO2. Further research unveiling the host targets of these effectors will shed light on the specific mode of action of the HopAO family in *P. savastanoi* pv. *savastanoi*.

Deletion of single effector genes does not often lead to a noticeable loss of virulence as measured by the attenuation of bacterial growth and symptom development in infected tissue (Hauck et al., 2003). However, a *P. savastanoi* pv. *savastanoi* NCPPB 3335 mutant defective in *hopAO1*, but encoding a wild-type *hopAO2*, showed a reduced ability to grow in olive plants (**Figure 6**). These results demonstrate that the Δ*hopAO1* mutant is affected in growth and knot formation in both micropropagated and lignified olive plants, and suggest that HopAO1 and HopAO2 do not show fully redundant functions. Similar results were previously reported for a *P. syringae* pv. *tomato* DC3000 *hopAO1* mutant in tomato leaves (Espinosa et al., 2003). Moreover, necrosis of the knot tissues induced by the NCPPB 3335 Δ*hopAO1* mutant was higher than that observed in plants infected with the wild-type strain (**Figure 6**). Additionally, the increased necrosis of knot tissues induced by the Psv48ΔAB NCPPB 3335 derivative (Bardaji et al., 2011) is fully restored by the expression of HopAO1, indicating that this effector is responsible for the inhibition of plant cell death during the interaction of Psv with its host plant. Other reports showing an effect of T3E on the induction of necrosis associated with both compatible and incompatible interactions with plants have been previously described, as is the case for the *P. syringae* pv. *tomato* DC3000 HopN1 (López-Solanilla et al., 2004). The regulation of plant cell death is of central importance during plant-pathogen interactions, as the host resistance response is associated with features involving cell death. Today, it is known how the host integrates signals to activate salicylic acid and reactive oxygen pathways that orchestrate cell death (Dickman and Fluhr, 2013). Here, we report that HopAO1 and HopAO2 activities are involved in the suppression of plant cell death both in an interaction involving immunity (together with the suppression of ROS production) and during the interaction rending disease.

The functional studies of the HopAO family members have been restricted to the analysis of HopAO1 homologs of strains isolated from herbaceous host (Bretz et al., 2003; Espinosa et al., 2003; Underwood et al., 2007; Macho et al., 2014; Üstün et al., 2016). However, there is no evidence, to date, of functional characterization of HopAO1 homologs of strains isolated from woody host. On the other hand this is the first report describing the behavior as a actively deployed effector of a HopAO2 homolog, a putative tyrosine phosphatase family member with only 33% identity with HopAO1. This work establishes NCPPB 3335 HopAO1 and HopAO2 effectors as modulators of the immune response during the interaction of *P. savastanoi* pv. *savastanoi* with plants.

## Author Contributions

MC-O carried out experimental design, experimental work, data analysis, and writing of the manuscript. AM-P carried out experimental work and data analysis. CR and EL-S conceived the idea and contributed to the experimental design, data analysis, interpretation of results and writing manuscript. All authors read and approved the final draft.

## Conflict of Interest Statement

The authors declare that the research was conducted in the absence of any commercial or financial relationships that could be construed as a potential conflict of interest.

## Footnotes

1 http://www.geneious.com

2 http://www.geneious.com

## Supplementary Material

**FIGURE S1 | Amino acid sequence alignment of the *Pseudomonas savastanoi* pv. *savastanoi* NCPPB 3335 type III effectors HopAO1 and HopAO2.** Conserved Cys in the HopAO family is highlighted in grey.
