# Supplementary figures and images for: Suppression of Plant Immune Responses by the Pseudomonas savastanoi pv. savastanoi NCPPB 3335 Type III Effector Tyrosine Phosphatases HopAO1 and HopAO2
Source: Front Plant Sci. 2017 May 5;8:680. doi: 10.3389/fpls.2017.00680 (PMC5418354; doi:10.3389/fpls.2017.00680)

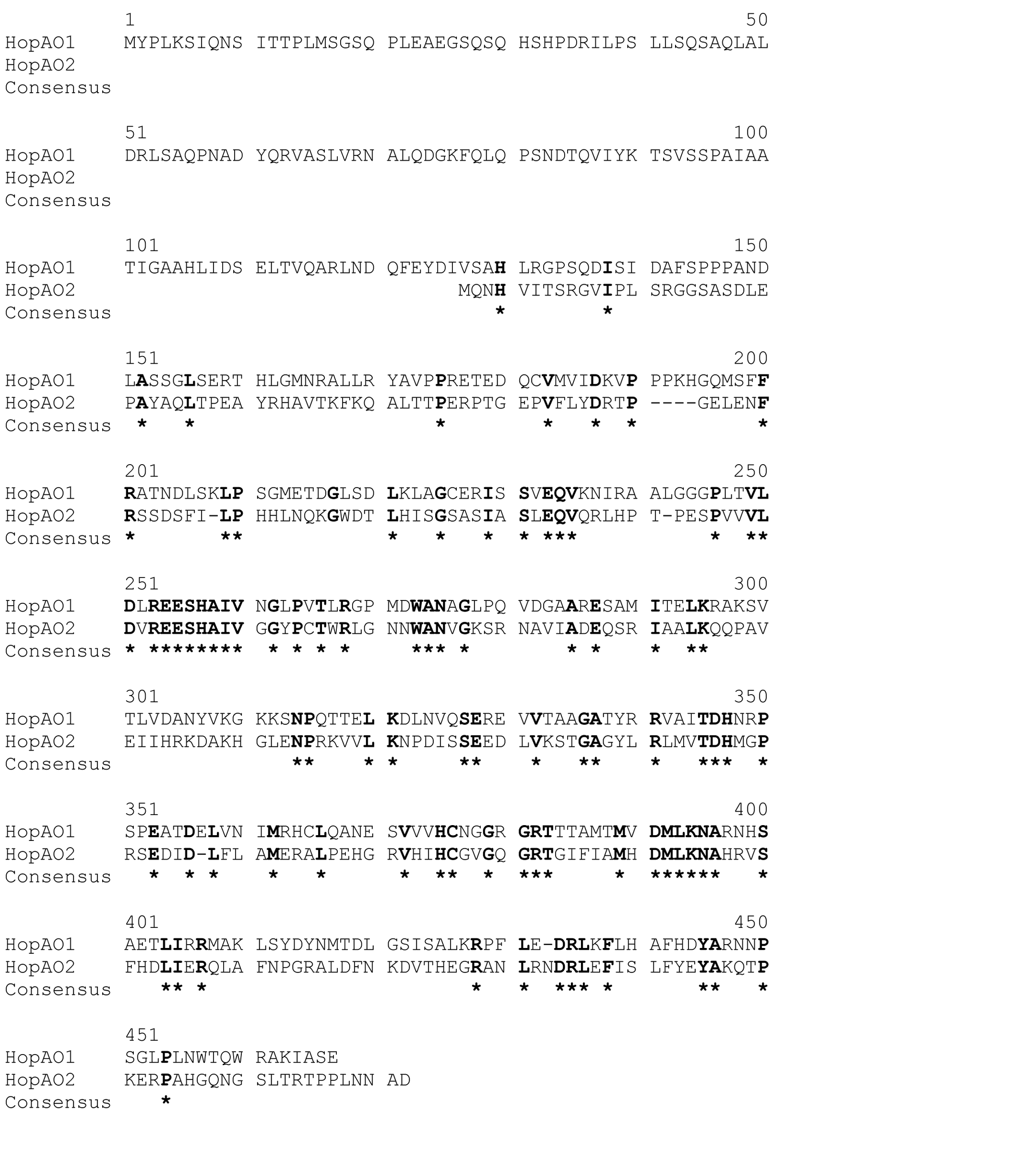

Supplement: Supplementary file 1 [file Image_1.TIF]
