# Supplementary material for: Suppression of Plant Immune Responses by the Pseudomonas savastanoi pv. savastanoi NCPPB 3335 Type III Effector Tyrosine Phosphatases HopAO1 and HopAO2
Source: Front Plant Sci. 2017 May 5;8:680. doi: 10.3389/fpls.2017.00680 (PMC5418354; doi:10.3389/fpls.2017.00680)
Supplement: Supplementary file 2 [file Table_1.docx]

| **Table S1.** Strain information and accession numbers of the protein sequences used for the construction of the phylogenetic trees shown in Figure 1. | | | | | | | | |
| --- | --- | --- | --- | --- | --- | --- | --- | --- |
| **Pathovar** | **Strain** | **Abbreviated** | **PG^a^** | **Host** | **Trait^b^** | **Accession number^c^** | **Locus Tag** | **Reference** |
| **HopAO1** |  |  |  |  |  |  |  |  |
| aesculi | 2113 | Pae | 3 | Horse-chestnut | W | KWT38806.1 | AMC94_20030 | Nowell *et al.* (2016) |
| aesculi | 2250 | Pae | 3 | Horse-chestnut | W | WP_010217001.1 | na | Baltrus *et al.* (2011) |
| aesculi | 2279 | Pae | 3 | Horse-chestnut | W | KWT06972.1 | AL041_25565 | Nowell *et al.* (2016) |
| aesculi | 2306 | Pae | 3 | Horse-chestnut | W | KWT15247.1 | AL042_08320 | Nowell *et al.* (2016) |
| aesculi | 2315 | Pae | 3 | Horse-chestnut | W | KWT17638.1 | AL043_07235 | Nowell *et al.* (2016) |
| aesculi | 2329 | Pae | 3 | Horse-chestnut | W | KWT18166.1 | AL044_05405 | Nowell *et al.* (2016) |
| aesculi | 2336 | Pae | 3 | Horse-chestnut | W | KWT35155.1 | AL045_26155 | Nowell *et al.* (2016) |
| aesculi | 0893-23 | Pae | 3 | Horse-chestnut | W | EGH05151.1 | PSYAE_24952 | Baltrus *et al.* (2011) |
| aesculi | ICMP 8947 | Pae | 3 | Horse-chestnut | W | KPW10093.1 | ALO90_200017 | Thakur *et al.* (2016) |
| aesculi | NCPPB 3681 | Pae | 3 | Horse-chestnut | W | KPW10093.1 | ALO90_200017 | Green *et al.* (2010) |
| alisalensis | ICMP 15200 | Pal | 5 | Radish | H | KPW17942.1 | ALO83_04035 | Baltrus *et al.* (2011) |
| berberidis | ICMP 4116 | Pbb | 1 | Barberry | WH | KPW49704.1 | ALO86_04163 | Thakur *et al.* (2016) |
| broussonetiae | ICMP 13650 | Pbr | 3 | Paper mulberry | WH | KWT12668.1 | AL047_00700 | Thakur *et al.* (2016) |
| fraxini | ICMP 7711 | Psf | 3 | Ash tree | W | na | na | Thakur *et al.* (2016) |
| mori | MAFF 301020 | Pmo | 3 | White mulberry | WH | EGH27329.1 | PSYMO_39895 | Baltrus *et al.* (2011) |
| orizae | 1_6 | Por | 4 | Rice | H | EGI01384.1 | POR16_06511 | Reinhardt *et al.* (2009) |
| papulans | CFBP 1754 | Psp | 2 | Apple | WH | KPY23606.1 | ALO65_02853 | Bartoli *et al.* (2015) |
| philadelphi | ICMP 8903 | Ppd | 1 | Mock orange | WH | KPY16031.1 | ALO54_02915 | Thakur *et al.* (2016) |
| porri | ICMP 8961 | Ppo | 4 | Leek | H | KPY25151.1 | ALO89_04452 | Thakur *et al.* (2016) |
| porri | LMG 28495 | Ppo | 4 | Leek | H | KOP51521.1 | OX88_25490 | Rombouts *et al.* (2016) |
| savastanoi | DAPP-PG722 | Psv | 3 | Olive tree | W | WP_002556090.1 | na | Moretti *et al.* (2014) |
| savastanoi | ICMP 4352 | Psv | 3 | Olive tree | W | KPB20516.1 | AC519_0598 | Thakur *et al.* (2016) |
| savastanoi | NCPPB 3335 | Psv | 3 | Olive tree | W | YP_006961588.1 | PSPSV_B0010 | Rodríguez-Palenzuela *et al.* (2010) |
| tomato | DC3000 | Pto | 1 | Tomato | H | NP_794465.1 | PSPTO_4722 | Buell *et al.* (2003) |
| **HopAO2** |  |  |  |  |  |  |  |  |
| actinidiae | CRAFRU 8.43 | Pan | 1 | Kiwifruit | W | WP_017684870.1 | na | Marcelletti *et al.* (2011) |
| actinidiae | MAFF 302091 | Pan | 1 | Kiwifruit | W | EGH67218.1 | PSYAC_20366 | Baltrus *et al.* (2011) |
| actinidiae | NCPPB 3739 | Pan | 1 | Kiwifruit | W | WP_017684870.1 | na | Marcelletti *et al.* (2011) |
| actinidiae | NCPPB 3871 | Pan | 1 | Kiwifruit | W | WP_017684870.1 | na | Marcelletti *et al.* (2011) |
| actinidiae | ICMP 18884 | Pan | 1 | Kiwifruit | W | AKT28618.1 | IYO_003720 | Templeton *et al.* (2015) |

| **Table S1.** Continued | |  |  |  |  |  |  |  |
| --- | --- | --- | --- | --- | --- | --- | --- | --- |
| **Pathovar** | **Strain** | **Abbreviated** | **PG^a^** | **Host** | **Trait^b^** | **Accession number^c^** | **Locus Tag** | **Reference** |
| actinidiae | ICMP 9617 | Pan | 1 | Kiwifruit | W | KCU99311.1 | A250_04298 | McCann *et al.* (2013) |
| berberidis | ICMP 4116 | Pbb | 1 | Barberry | WH | KPW45431.1 | ALO86_200168 | Thakur *et al.* (2016) |
| caricapapayae | ICMP 2855 | Pca | 6 | Papaya | WH | KPW61170.1 | ALO80_03646 | Thakur *et al.* (2016) |
| cerasicola | ICMP 17524 | Pce | 3 | cherry | W | KPX02378.1 | ALO50_01699 | Nowell *et al.* (2016) |
| ciccaronei | ICMP 5710 | Pcc | 3 | Carob tree | WH | KPW68734.1 | ALO78_01725 | Thakur *et al.* (2016) |
| coriandricola | ICMP 12471 | Pcd | 5 | Cilantro | H | KPW79583.1 | ALO76_01524 | Thakur *et al.* (2016) |
| cunninghamiae | ICMP 11894 | Pcu | 3 | Chinese fir | W | WP_057455977.1 | na | Thakur *et al.* (2016) |
| fraxini | ICMP 7711 | Psf | 3 | Ash tree | W | na | na | Thakur *et al.* (2016) |
| ficuserectae | ICMP 7848 | Pfi | 3 | Inubiwa | WH | KPX38837.1 | ALO69200116 | Thakur *et al.* (2016) |
| glycinea | B076 | Pgy | 3 | Soybean | H | EFW77747.1 | PsgB076_26910 | Qi *et al.* (2011) |
| glycinea | ICMP 2189 | Pgy | 3 | Soybean | H | WP_032704352.1 | na | Thakur *et al.* (2016) |
| helianthi | ICMP 4531 | Phe | 6 | Sunflower | H | KPX50393.1 | ALO68_200225 | Thakur *et al.* (2016) |
| hibisci | ICMP 9623 | Phi | 3 | Chinese hibiscus | WH | KPX58891.1 | ALO67_200085 | Thakur *et al.* (2016) |
| maculicola | ES4326 | Pma | 1 | Cauliflower | H | EGH63066.1 | PMA4326_30047 | Baltrus *et al.* (2011) |
| meliae | ICMP 6289 | Pml | 3 | Chinaberry | W | KPX86922.1 | ALO64_03355 | Bartoli *et al.* (2015) |
| mori | MAFF 301020 | Pmo | 3 | White mulberry | WH | EGH25072.1 | PSYMO_27904 | Baltrus *et al.* (2011) |
| nerii | ICMP 16943 | Psn | 3 | Oleander | W | KPY02150.1 | ALO61_00905 | Thakur *et al.* (2016) |
| philadelphi | ICMP 8903 | Ppd | 1 | Mock orange | WH | KPY16031.1 | ALO54_02915 | Thakur *et al.* (2016) |
| retacarpa | CECT 4861 | Psr | 3 | Broom | W | KPY46553.1 | ALO49_200281 | Thakur *et al.* (2016) |
| savastanoi | DAPP-PG722 | Psv | 3 | Olive tree | W | na | na | Moretti *et al.* (2014) |
| savastanoi | ICMP 4352 | Psv | 3 | Olive tree | W | KPY71942.1 | ALO58_02077 | Thakur *et al.* (2016) |
| savastanoi | NCPPB 3335 | Psv | 3 | Olive tree | W | EFH97024.2 | PSA3335_5047 | Rodríguez-Palenzuela *et al.* (2010) |
| savastanoi | PseNe 107 | Psv | 3 | Olive tree | W | na | na | Bartoli *et al.* (2015) |
| tomato | K40 | Pto | 1 | Tomato | H | ZP_07253402 | PsyrptK_010100017896 | Cai *et al.* (2011) |
| tomato | Max13 | Pto | 1 | Tomato | H | ZP_07235023 | PsyrptM_010100028414 | Cai *et al.* (2011) |

^a^PG, phylogroup

^b^Trait: W, woody organ (trunk, stem and/or branches) of woody host; H, herbaceous host; WH, herbaceous organ (leaf) of woody host

^c^Obtained from GenBank; na, not available

**REFERENCES**

Baltrus, D.A., Nishimura, M.T., Romanchuk, A., Chang, J.H., Mukhtar, M.S. and Cherkis, K. (2011) Dynamic evolution of pathogenicity revealed by sequencing and comparative genomics of 19 Pseudomonas syringae isolates. *PLoS Pathog.* 7.

Bartoli, C., Carrere, S., Lamichhane, J.R., Varvaro, L. and Morris, C.E. (2015) Whole-Genome Sequencing of 10 Pseudomonas syringae Strains Representing Different Host Range Spectra. *Genome Announc.* 3.

Buell, C. R., Joardar, V., Lindeberg, M., Selengut, J., Paulsen, I. T. and Gwinn, M. L. (2003) The complete genome sequence of the Arabidopsis and tomato pathogen Pseudomonas syringae pv. tomato DC3000. *Proc Natl Acad Sci U S A,* **100**.

Cai, R., Lewis, J., Yan, S., Liu, H., Clarke, C.R., Campanile, F.*, et al****.*** (2011) The Plant Pathogen Pseudomonas syringae pv. tomato Is Genetically Monomorphic and under Strong Selection to Evade Tomato Immunity. *PLoS Pathog.* **7,** e1002130.

Green, S., Studholme, D.J., Laue, B.E., Dorati, F., Lovell, H., Arnold, D.*, et al.* (2010) Comparative genome analysis provides insights into the evolution and adaptation of *Pseudomonas syringae* pv. aesculi on *Aesculus hippocastanum*. *PLoS One,* **5,** e10224.

Marcelletti, S., Ferrante, P., Petriccione, M., Firrao, G. and Scortichini, M. (2011) Pseudomonas syringae pv. actinidiae Draft Genomes Comparison Reveal Strain-Specific Features Involved in Adaptation and Virulence to Actinidia Species. *PLoS ONE,* **6,** e27297.

McCann, H.C., Rikkerink, E.H. A., Bertels, F., Fiers, M., Lu, A., Rees-George, J.*, et al.* (2013) Genomic Analysis of the Kiwifruit Pathogen Pseudomonas syringae pv. actinidiae Provides Insight into the Origins of an Emergent Plant Disease. *PLOS Pathog.* 9**,** e1003503.

Moretti, C., Cortese, C., Passos da Silva, D., Venturi, V., Ramos, C., Firrao, G.*, et al****.*** (2014) Draft Genome Sequence of Pseudomonas savastanoi pv. savastanoi Strain DAPP-PG 722, Isolated in Italy from an Olive Plant Affected by Knot Disease. *Genome Announcements,* 2**,** e00864-00814.

Nowell, R.W., Laue, B.E., Sharp, P. M. and Green, S. (2016) Comparative genomics reveals genes significantly associated with woody hosts in the plant pathogen Pseudomonas syringae. *Mol. Plant Pathol.* **17,** 1409-1424.

Qi, M., Wang, D., Bradley, C.A. and Zhao, Y. (2011) Genome Sequence Analyses of Pseudomonas savastanoi pv. glycinea and Subtractive Hybridization-Based Comparative Genomics with Nine Pseudomonads. *PLoS One,* 6, e16451.

Reinhardt, J.A., Baltrus, D.A., Nishimura, M.T., Jeck, W. R., Jones, C.D. and Dangl, J.L. (2009) De novo assembly using low-coverage short read sequence data from the rice pathogen *Pseudomonas syringae* pv. oryzae. *Genome Res.* 19, 294-305.

Rodríguez-Palenzuela, P., Matas, I.M., Murillo, J., López-Solanilla, E., Bardaji, L., Pérez-Martínez, I.*, et al.* (2010) Annotation and overview of the *Pseudomonas savastanoi* pv. savastanoi NCPPB 3335 draft genome reveals the virulence gene complement of a tumour-inducing pathogen of woody hosts. *Environ. Microbiol.* 12, 1604-1620.

Rombouts, S., Van Vaerenbergh, J., Volckaert, A., Baeyen, S., De Langhe, T., Declercq, B.*, et al.* (2016) Isolation and characterization of Pseudomonas syringae pv. porri from leek in Flanders. *Europ. J. of Plant Pathol.* 144, 185-198.

Templeton, M.D., Warren, B.A., Andersen, M.T., Rikkerink, E.H.A. and Fineran, P.C**.** (2015) Complete DNA Sequence of *Pseudomonas syringae* pv. actinidiae, the Causal Agent of Kiwifruit Canker Disease. *Genome Announc.* 3**,** e01054-01015.

Thakur, S., Weir, B.S. and Guttman, D.S. (2016) Phytopathogen Genome Announcement: Draft Genome Sequences of 62 Pseudomonas syringae Type and Pathotype Strains. *Mol. Plant-Microbe Interact.* 29**,** 243-246.
