# Supplementary material for: Suppression of Plant Immune Responses by the Pseudomonas savastanoi pv. savastanoi NCPPB 3335 Type III Effector Tyrosine Phosphatases HopAO1 and HopAO2
Source: Front Plant Sci. 2017 May 5;8:680. doi: 10.3389/fpls.2017.00680 (PMC5418354; doi:10.3389/fpls.2017.00680)
Supplement: Supplementary file 3 [file Table_2.doc]

| **Table S2.** Bacterial strains used in this study. | | | | |
| --- | --- | --- | --- | --- |
| **Strains^a^** | **Relevant characteristics** | | | **References** |
| ***E. coli*** | | | |  |
| DH5α | | *F -, ϕ80dlacZ M15, (lacZYA-argF) U169, deoR, recA1, endA, hsdR17 (rk - mk -), phoA, supE44, thi-1, gyrA96, relA1.* | | (Hanahan, 1983) |
| XL1 Blue | | *hsdR17, supE44, recA1, endA1, gyrA46, thi, relA1, lac/ F*′ [*proAB^+^, lacI^q^, lacZ M15::*Tn10 (Tc^R^)] | | (Bullock *et al*., 1987) |
| GM2929 | | *F -, ara-14, leuB6, thi-1, tonA31, lacY1, tsx-78, galK2, galT22, glnV44, hisG4, rpsL136, xyl-5,mtl-1, dam13::Tn9, dcm-6, mcrB1, hsdR2, mcrA, recF143*. (Sp^R^ Cm^R^)*.* | | (Palmer and Marinus, 1994) |
| BL21(DE3) | | *F-, ompT, gal, dcm, lon, hsdSB* (*r_B_^-^ m_B_^-^*)*, λ* (*DE3* [*lacI, lacUV5-T7, gene 1, ind1, sam7, nin5*]) | | (Studier *et al.*, 1990) |
| ***Pseudomonas*** | |  | |  |
| *P. fluorescens* 55 [pLN18] (Pf) | | Containing 25 kb *P. syringae* pv. syringae 61 *hrc/hrp* cluster with *shcA* and *hopPsyA* replaced by an *nptII* cassette (Km^R^) . | | (Jamir *et al*., 2004) |
| *P. savastanoi* pv. nerii (Psn) | | |  |  |
| 2 | Isolated from oleander | | | (Matas *et al*., 2009) |
| 519 | Isolated from oleander | | | (Surico *et al*., 1985) |
| *P. savastanoi* pv. savastanoi (Psv) | | | |  |
| NCPPB 3335 | Isolated from olive | | | (Pérez-Martínez *et al*., 2007) |
| NCPPB 3335-T3 | Type III secretion mutant (Km^R^) | | | (Pérez-Martinez *et al*., 2010) |
| NCPPB 3335 ∆*hrpL* | *hrpL* mutant derived from NCPPB 3335 (Km^R^) | | | (Matas IM *et al*., 2014) |
| Psv48 ΔAB | NCPPB 3335 cured of pPsv48A and pPsv48B | | | (Bardaji *et al.*, 2011) |
| Δ*hopAO1* | *hopAO1* mutant derived from NCPPB 3335 (Km^R^) | | | This study |
| DAPP-PG722 | Isolated from olive | | | (Hosni *et al*., 2011) |
| PVFi-1 | Isolated from olive | | | (Iacobellis *et al*., 1993) |
| IMC-1 | Isolated from olive | | | (Matas *et al*., 2009) |
| IMC-2 | Isolated from olive | | | (Matas *et al*., 2009) |
| ICMP 4352 | Isolated from olive | | | (Thakur *et al*., 2016) |
| CFBP 2074 | Isolated from olive | | | (Penyalver *et al*., 2000) |
| CFBP 71 | Isolated from olive | | | (Penyalver *et al*., 2000) |
| IVIA 1628-3 | Isolated from olive | | | (Penyalver *et al*., 2000) |
| IVIA 1629-1a | Isolated from olive | | | (Penyalver *et al*., 2000) |
| IVIA 1624-1b | Isolated from olive | | | (Penyalver *et al*., 2000) |
| IVIA 1637-a | Isolated from olive | | | (Penyalver *et al*., 2000) |
| IVIA 1637-B3 | Isolated from olive | | | (Penyalver *et al*., 2000) |
| IVIA 1649-1 | Isolated from olive | | | (Penyalver *et al*., 2000) |
| IVIA1651-C15 | Isolated from olive | | | (Penyalver *et al*., 2000) |
| IVIA 1657-A2 | Isolated from olive | | | (Penyalver *et al*., 2000) |
| IVIA 1657-B8 | Isolated from olive | | | (Penyalver *et al*., 2000) |
| NCPPB 2327 | Isolated from olive | | | (Penyalver *et al*., 2000) |
| NCPPB 1342 | Isolated from olive | | | (Penyalver *et al*., 2000) |
| NCPPB 1344 | Isolated from olive | | | (Penyalver *et al*., 2000) |
| NCPPB 1479 | Isolated from olive | | | (Penyalver *et al*., 2000) |
| NCPPB 1506 | Isolated from olive | | | (Penyalver *et al*., 2000) |
| NCPPB 64 | Isolated from olive | | | (Penyalver *et al.*, 2000) |
| **Strains^a^** | **Relevant characteristics** | | | **References** |
| CFBP 1020 | Isolated from olive | | | (Penyalver *et al.*, 2000) |
| C2.01 | Isolated from olive | | | (Pérez-Martínez *et al.*, 2007) |
| C2.01 | Isolated from olive | | | (Pérez-Martínez *et al*., 2008) |
| B15.00 | Isolated from olive | | | (Pérez-Martínez *et al.*, 2008) |
| C1.01 | Isolated from olive | | | (Pérez-Martínez *et al*., 2008) |
| C3.01 | Isolated from olive | | | (Pérez-Martínez *et al.*, 2008) |
| IVIA 2733-1a, | Isolated from olive | | | (Quesada *et al*., 2008) |
| IVIA 2743-3 | Isolated from olive | | | (Quesada *et al.*, 2008) |
| ITM317 | Isolated from olive | | | (Surico *et al*., 1985) |
| *P. syringae* pv. actinidiae (Pan) | | | |  |
| KACC10594 | Isolated from kiwi | | | (Rees-George *et al.*, 2010) |
| *P. syringae* pv. dendropanacis (Pde) | | | |  |
| CFBP 3226 | Isolated from *Dendropanax* | | | (Gardan *et al.*, 1999) |
| *P. syringae* pv. eriobotryae (Per) | | | |  |
| CFBP 2343 | Isolated from loquat | | | (Gardan *et al.*, 1999) |
| *P. syringae* pv. glycinea (Pgy) | | | |
| NCPPB 1139 | Isolated from *Glycine javanica* | | | (Yamamoto *et al.*, 2000) |
| PG4180 | Isolated from soybean | | | (Mitchell, 1978) |
| *P. syringae* pv. lachrymans (Pla) | | | |  |
| CFBP 1644 | Isolated from cucumber | | | (Gardan *et al.*, 1999) |
| *P. syringae* pv. maculicola (Pma) | | | |  |
| CFBP1657 | Isolated from cauliflower | | | (Gardan *et al.*, 1999) |
| *P. syringae* pv. morsprunorum (Pmp) | | | |  |
| CFBP 2116 | Isolated from tart cherry | | | (Gardan *et al*., 1999) |
| *P. syringae* pv. myricae (Pmy) | | | |  |
| CFBP 2897 | Isolated from red bayberry | | | (Gardan *et al.*, 1999) |
| *P. syringae* pv. phaseolicola (Pph) | | | |  |
| 1448A | Isolated from bean | | | (Teverson, 1991) |
| 1449B | Isolated from *Lablab purpureus* | | | (Taylor et al., 1996) |
| *P. syringae* pv. sesami (Pse) | | | |  |
| CFBP 1671 | Isolated from sesame | | | (Gardan *et al*., 1999)  - |
| *P. syringae* pv. syringae (Psy) | | | |
| B728a | Isolated from bean | | | (Loper and Lindow, 1987) |
| FF5 | Isolated from ornamental pear | | | (Sundin and Bender, 1993) |
| *P. syringae* pv. tabaci (Pta) | | | |  |
| ATCC 11528 | Isolated from tobacco | | | (Studholme, 2011) |
| *P. syringae* pv. tomato (Pto) | | | |  |
| PT23 | Isolated from tomato | | | (Bender and Cooksey, 1986) |
| DC3000 | Isolated from tomato | | | (Cuppels, 1986) |
| DC3000D28E | *ΔhopU1-hopF2 ΔhopC1-hopH1*::FRT *ΔhopD1-hopR1*::FRT *ΔavrE-shcN ΔhopAA1-2-hopG1::*FRT *ΔhopI1 ΔhopAM1-1 ΔhopAF1*::FRT *ΔavrPtoB ΔavrPto ΔhopK1 ΔhopB1 ΔhopE1 ΔhopA1::*FRT *hopY1:*:FRT pDC3000A− pDC3000B− (Sp^R^) | | | (Cunnac *et al*., 2011) |

^a^Cm^R^, Km^R^, Sp^R^ and Rif^R^ indicate resistance to chloramphenicol, kanamycin, spectinomycin and rifampicin, respectively. ^b^ATCC, American Type Culture Collection.

**REFERENCES**

Bardaji, L., Perez-Martinez, I., Rodriguez-Moreno, L., Rodriguez-Palenzuela, P., Sundin, G. W., Ramos, C.*, et al.* (2011) Sequence and role in virulence of the three plasmid complement of the model tumor-inducing bacterium *Pseudomonas savastanoi* pv. savastanoi NCPPB 3335. *PLoS One,* 6: e25705.

Bender, C.L. and Cooksey, D.A. (1986) Indigenous plasmids in *Pseudomonas syringae* pv. tomato: conjugative transfer and role in copper resistance. *J. Bacteriol.* 165, 534–541.

Bullock, W.O., Fernandez, J.M. and Short, J.M. (1987) Xl1-Blue: a high efficiency plasmid transforming *recA Escherichia coli* strain with beta-galactosidase selection. *Biotechniques* 5, 376-378.

Cunnac, S., Chakravarthy, S., Kvitko, B. H., Russell, A. B., Martin, G.B. and Collmer, A. (2011) Genetic disassembly and combinatorial reassembly identify a minimal functional repertoire of type III effectors in *Pseudomonas syringae*. *Proc. Natl. Acad. Sci. U S A,* 108, 2975-2980.

Cuppels, D.A. (1986) Generation and Characterization of Tn5 Insertion Mutations in *Pseudomonas syringae* pv. tomato. *Appl. Environ. Microbiol.* **51,** 323-327.

Gardan, L., Shafik, H., Belouin, S., Broch, R., Grimont, F. and Grimont, P.A.D. (1999) DNA relatedness among the pathovars of *Pseudomonas syringae* and description of *Pseudomonas tremae* sp. nov. and *Pseudomonas cannabina* sp. nov. (*ex* Sutic and Dowson 1959). *Int. J. Syst. Bacteriol.* 49, 469-478.

Hanahan, D. (1983) Studies on transformation of *Escherichia coli* with plasmids. *J. Mol. Biol.* 166, 557-580.

Hosni, T., Moretti, C., Devescovi, G., Suárez-Moreno, Z.R., Fatmi, M.B., Guarnaccia, C.*, et al.* (2011) Sharing of quorum-sensing signals and role of interspecies communities in a bacterial plant disease. *ISME J.* **5,** 1857-1870.

Iacobellis, N.S., Sisto, A. and Surico, G. (1993) Occurrence of unusual strains of *Pseudomonas syringae* subsp. savastanoi on olive in central Italy. *Bull. OEPP.* 23, 429–435.

Jamir, Y., Guo, M., Oh, H.S., Petnicki-Ocwieja, T., Chen, S., Tang, X.*, et al.* (2004) Identification of *Pseudomonas syringae* type III effectors that can suppress programmed cell death in plants and yeast. *Plant J.* 37, 554-565.

Loper, J.E. and Lindow, S.E. (1987) Lack of evidence for in situ fluorescent pigment production by *Pseudomonas syringae* pv. syringae on bean leaf surfaces. *Phytopathol.* 77, 1449-1454.

Matas IM, Castañeda-Ojeda MP, Aragón IM, Antúnez-Lamas M, Murillo J, Rodríguez-Palenzuela P*, et al.* (2014) Translocation and functional analysis of *Pseudomonas savastanoi* pv. *savastanoi* NCPPB 3335 type III secretion system effectors reveals two novel effector families of the *Pseudomonas syringae* complex. *Mol. Plant Microbe Interact.* 27, 424-436.

Matas, I.M., Pérez-Martínez, I., Quesada, J.M., Rodríguez-Herva, J.J., Penyalver, R. and Ramos, C. (2009) *Pseudomonas savastanoi* pv. savastanoi contains two *iaaL* paralogs, one of which exhibits a variable number of a trinucleotide (TAC) tandem repeat. *Appl. Environ. Microbiol.* 75, 1030-1035.

Mitchell, R.E. (1978) Halo blight of beans: Toxin production by several *Pseudomonas phaseolicola* isolates. *Physiol. Plant Pathol.* 13, 37-49.

Palmer, B.R. and Marinus, M.G**.** (1994) The dam and dcm strains of *Escherichia coli*--a review. *Gene,* 143, 1-12.

Penyalver, R., García, A., Ferrer, A., Bertolini, E. and López, M.M. (2000) Detection of *Pseudomonas savastanoi* pv. *savastanoi* in olive plants by enrichment and PCR. *Appl. Environ. Microbiol****.*** 66, 2673-2677.

Pérez-Martinez, I., Rodriguez-Moreno, L., Lambertsen, L., Matas, I.M., Murillo, J., Tegli, S.*, et al.* (2010) Fate of a *Pseudomonas savastanoi* pv. *savastanoi* type III secretion system mutant in olive plants (Olea europaea L.). *Appl. Environ. Microbiol.* 76, 3611-3619.

Pérez-Martínez, I., Rodríguez-Moreno, L., Matas, I.M. and Ramos, C. (2007) Strain selection and improvement of gene transfer for genetic manipulation of *Pseudomonas savastanoi* isolated from olive knots. *Res. Microbiol.* 158, 60-69.

Pérez-Martínez, I., Zhao, Y., Murillo, J., Sundin, G.W. and Ramos, C. (2008) Global genomic analysis of *Pseudomonas savastanoi* pv. savastanoi plasmids. *J. Bacteriol.* 190, 625-635.

Quesada, J.M., Pérez-Martínez, I., Ramos, C., López, M.M. and Penyalver, R. (2008) IS53: an insertion element for molecular typing of *Pseudomonas savastanoi* pv. *savastanoi.* *Res. Microbiol.* 159, 207-215.

Rees-George, J., Vanneste, J.L., Cornish, D.A., Pushparajah, I.P.S., Yu, J., Templeton, M. D.*, et al.* (2010) Detection of *Pseudomonas syringae* pv. actinidiae using polymerase chain reaction (PCR) primers based on the 16S–23S rDNA intertranscribed spacer region and comparison with PCR primers based on other gene regions. *Plant Patholol.* 59, 453-464.

Studholme, D.J. (2011) Application of high-throughput genome sequencing to intrapathovar variation in *Pseudomonas syringae*. *Mol. Plant Pathol.* 12, 829-838.

Studier, F.W., Rosenberg, A.H., Dunn, J.J. and Dubendorff, J.W. (1990) Use of T7 RNA polymerase to direct expression of cloned genes. *Methods Enzymol.* 185, 60-89.

Surico, G., Iacobellis, N.S. and Sisto, A. (1985) Studies on the role of indole-3-acetic acid and cytokinins in the formation of knots on olive and oleander plants by *Pseudomonas syringae* pv. *savastanoi.* *Physiol. Plant Pathol.* 26, 309-320.

Taylor, J.D., Teverson, D.M., Allen, D.J. and Pastor-Corrales, M.A. (1996) Identification and origin of races of *Pseudomonas syringae* pv. phaseolicola from Africa and other bean growing areas. *Plant Pathol.* 45, 469-478.

Thakur, S., Weir, B.S. and Guttman, D.S. (2016) Phytopathogen Genome Announcement: Draft Genome Sequences of 62 *Pseudomonas syringae* Type and Pathotype Strains. *Mol. Plant-Microbe Interact.* 29, 243-246.

Teverson, D.M. (1991) Genetics of pathogenicity and resistance in the halo-blight disease of beans in Africa. Birmingham, UK: University of Birmingham.

Yamamoto, S., Kasai, H., Arnold, D.L., Jackson, R.W., Vivian, A. and Harayama, S. (2000) Phylogeny of the genus *Pseudomonas:* intrageneric structure reconstructed from the nucleotide sequences of *gyrB* and *rpoD* genes. *Microbiology,* 146, 2385-2394.
