# Supplementary material for: Suppression of Plant Immune Responses by the Pseudomonas savastanoi pv. savastanoi NCPPB 3335 Type III Effector Tyrosine Phosphatases HopAO1 and HopAO2
Source: Front Plant Sci. 2017 May 5;8:680. doi: 10.3389/fpls.2017.00680 (PMC5418354; doi:10.3389/fpls.2017.00680)
Supplement: Supplementary file 4 [file Table_3.doc]

**Table S3.** Plasmids used in this study.

| **Name** | **Description^a^** | **References** |
| --- | --- | --- |
| pGEM-T | Cloning vector containing ori F1 and *lac*Z (Ap^R^) | (*Promega Corp.*;Wisconsin, USA) |
| pGEM-T- KmFRT-*EcoR*I | Contains Km^R^ from pKD4 (Ap^R^ Km^R^) | (Zumaquero et al., 2010) |
| pPCO2 | pGEM-T derivates, contains 1.2 kb approx. on each side of the *hopAO1* gene (AER-0000610) (Ap^R^) | This study |
| pPCO2-Km | pGEM-T derivates, contains 1.2 kb approx. on each side of the *hopAO1* gene (AER-0000610) interrupted by the kanamycin resistance gen *nptII* (Ap^R^, Km^R^) | This study |
| pENTR/D/SD TOPO | Entry vector for Gateway cloning (Km^R^, Cm^R^) | (*Invitrogen Corp*.;  California, USA) |
| pENTR-*hopAO1* | pENTR/D/SD TOPO::AER-0000610 (Km^R^) | This study |
| pENTR-*hopAO2* | pENTR/D/SD TOPO::AER-0000328 (Km^R^) | This study |
| pCPP3234 | pVLT35::Gateway cassette-Cya fusion, broad-host-range vector containing *tac* promoter and *lacI*^q^ (Sp^R^, Str^R^, Cm^R^) | (Schechter et al., 2004) |
| pCYA-*hopAO1* | pCPP3234 expressing AER-0000610-Cya (Sp^R^, Str^R^) | This study |
| pCYA-*hopAO2* | pCPP3234 expressing AER-0000328-Cya (Sp^R^, Str^R^) | This study |
| pCPP5040 | pML123::Gateway cassette, broad-host-range vector allowing for constitutive expression of inserts fused to a C-terminal HA tag from the *nptII* promoter (Gm^R^, Cm^R^) | (Lopez-Solanilla *et al*., 2004) |
| pEXP-*hopAO1* | pCPP5040 expressing AER-0000610-HA  tag (Gm^R^) | This study |
| pEXP-*hopAO2* | pCPP5040 expressing AER-0000328-HA  tag (Gm^R^) | This study |
| pDEST42 | pET Gateway^TM^ compatible vector allowing for T7-regulated expression of a protein with a C-terminal His_6_-V5 tag, Ap^R^ | (*Invitrogen Corp*.;  California, USA) |
| pPUR-*hopAO1* | pDEST42 expressing AER-0000610-6His tag (Ap^R^) | This study |
| pPUR-*hopAO1*-Cys_376_ | pDEST42 expressing AER-0000610-Cys_376_-6His tag (Ap^R^) | This study |

^a^Ap^R^, Gm^R^, Cm^R^, Km^R^, Sp^R^ and Str^R^ indicate resistance to ampicillin, gentamicin, chloramphenicol, kanamycin, spectinomycin and streptomycin, respectively. AER numbers indicate ASAP identification numbers for Psv NCPPB 3335 genes (http://www.genome.wisc.edu/tools/asap.htm). *Cya*, catalytic domain of *Bordetella pertusis* adenilate cyclase; HA tag, Influenza hemagglutinin peptide YPYDVPDYA.

**REFERENCES**

Lopez-Solanilla, E., Bronstein, P.A., Schneider, A.R. and Collmer, A. (2004). HopPtoN is a *Pseudomonas syringae* Hrp (type III secretion system) cysteine protease effector that suppresses pathogen-induced necrosis associated with both compatible and incompatible plant interactions. *Mol. Microbiol.* 54, 353-365.

Schechter, L.M., Roberts, K.A., Jamir, Y., Alfano, J.R. and Collmer, A. (2004). *Pseudomonas syringae* type III secretion system targeting signals and novel effectors studied with a Cya translocation reporter. *J. Bacteriol.* 186, 543-555.

Zumaquero, A., Macho, A.P., Rufian, J.S. and Beuzon, C.R. (2010). Analysis of the role of the type III effector inventory of *Pseudomonas syringae* pv. *phaseolicola* 1448a in interaction with the plant. *J. Bacteriol.* 192, 4474-4488.
