# Supplementary material for: Suppression of Plant Immune Responses by the Pseudomonas savastanoi pv. savastanoi NCPPB 3335 Type III Effector Tyrosine Phosphatases HopAO1 and HopAO2
Source: Front Plant Sci. 2017 May 5;8:680. doi: 10.3389/fpls.2017.00680 (PMC5418354; doi:10.3389/fpls.2017.00680)
Supplement: Supplementary file 5 [file Table_4.DOCX]

**Table S4.** Primers used in this study.

| **Name^a^** | **Sequence** |  | **Use** |
| --- | --- | --- | --- |
| HopAO1 F-96 | TCTCAGTCACAGCATTCC | Probe construction for dot blot analysis  and mutant verification | |
| HopAO1 R-366 | GCTTACGATGTCGTACTC |  |  |
| HopAO2-F82 | CGCAGTTGACTCCCGAAG | Probe construction for dot blot analysis  and mutant verification | |
| HopAO2-R469 | GAGATTCTTCTCGTACATC |  |  |
| Km R-768 | TTGCATCAGCCATGATGG | Probe construction for mutant verification | |
| Oligo P1 | GTGTAGGCTGGAGCTGCTTC |  |  |
| hopAO1-F | TGGTTGTGAACGCATTTCCTC | qRT-PCR | |
| hopAO1-R | GCATGAGATTCTTCGCGCA |  |  |
| hopAO2-F | TGCGTTTGATGGTGACCGA | qRT-PCR | |
| hopAO2-R | ACCGCAATGGATATGTACCCG |  |  |
| TA-HopAO1 F | TGTCGCTTAAGATCCAGC | Amplification for mutant construction | |
| HopAO1 R-93 | CCCTATAGTGAGTCGGATCCG  TTCCGAAGAATCAGAAC |  |  |
| TD-HopAO1-F20 | GGATCCGACTCACTATAGG  GACGGATGCCGAGGTTTG |  |  |
| TD-HopAO1 R | ACACGGTATGTACGTAGG |  |  |
| TOPO-610-F | CACCATGTATCCCCTGAAATCT | Amplifications for constructions used in translocation assays and heterologous expression experiments using Gateway technology | |
| TOPO-610-R | TTCTGACGCTATTTTTGC |  |  |
| TOPO-328-F | CACCATGCCGAAATTTCCGTCA |  |  |
| TOPO-328-R | GTCAGCGTTGTTGAGAGG |  |  |
| CS-hopAO1-F | GAGTCGGTAGTTGTGCACTCT  AACGGCGGTCGC | Construction of mutant in catalytic  domain | |
| CS-hopAO1-R | GCGACCGCCGTTAGAGTGC  ACAACTACCGACTC |  |  |
| CS-hopAO1-C | AGTCGGTAGTTGTGCACTC |  |  |
| CS-hopAO2-F | GGTCGGGTACATATCCATTCC  GGTGTCGGG | Construction of mutant in catalytic domain | |
| CS-hopAO2-R | CCCGACACCGGAATGGATATGTACCCGACC |  |  |
| CS-hopAO2-C | GTCGGGTACATATCCATTC |  |  |
| Cya | CAATCAGGCTGGTGGAATGG | Sequencing for construction verification | |

^a^ F and R, forward and reverse primer, respectively. Numbers included after F and R in primers names correspond to the hybridization position of the 3´ end of the primer in the corresponding ORF sequence.
