# Supplementary material for: Suppression of Plant Immune Responses by the Pseudomonas savastanoi pv. savastanoi NCPPB 3335 Type III Effector Tyrosine Phosphatases HopAO1 and HopAO2
Source: Front Plant Sci. 2017 May 5;8:680. doi: 10.3389/fpls.2017.00680 (PMC5418354; doi:10.3389/fpls.2017.00680)
Supplement: Supplementary file 6 [file Table_5.DOCX]

**Table S5.** Occurrence of consensus Hrp-box sequences upstream of *hopAO1* and *hopAO2* genes from *P. savastanoi* pv. *savastanoi* NCPPB 3335.

| **Gene name** | **Hrp-box position^a^** | **Hrp-box sequence** |
| --- | --- | --- |
| CONSENSUS^b^ |  | **BGGAACYHNNNNNNNNNNNNNNNCCACNHAG** |
| *hopAO1* | -86 to -55 | CGGAACCCCACAAGCATTTAAGACCACGTAT |
| *hopAO2* | -60 to -29 | TGGAACCGTTGGAGCCTATGCGCCCACGAAA |

^a^Coordinates are relative to the annotated start codon of the coding sequences.
